# Autophagy differentially regulates tissue tolerance of distinct target organs in graft-versus-host disease models

**DOI:** 10.1172/JCI167369

**Published:** 2024-03-01

**Authors:** Katherine Oravecz-Wilson, Emma Lauder, Austin Taylor, Laure Maneix, Jeanine L. Van Nostrand, Yaping Sun, Lu Li, Dongchang Zhao, Chen Liu, Pavan Reddy

**Affiliations:** 1 Department of Internal Medicine, Division of Hematology and Oncology, University of Michigan, Rogel Cancer Center, Ann Arbor, Michigan, USA.; 2 Dan L. Duncan Comprehensive Cancer Center and; 3 Department of Molecular and Cellular Biology, Baylor College of Medicine, Houston, Texas, USA.; 4 Department of Pathology, Yale School of Medicine, Yale University, New Haven, Connecticut, USA.

**Keywords:** Immunology, Transplantation, Autophagy

## Abstract

Tissue-intrinsic mechanisms that regulate severity of systemic pathogenic immune-mediated diseases, such as acute graft-versus-host disease (GVHD), remain poorly understood. Following allogeneic hematopoietic stem cell transplantation, autophagy, a cellular stress protective response, is induced in host nonhematopoietic cells. To systematically address the role of autophagy in various host nonhematopoietic tissues, both specific classical target organs of acute GVHD (intestines, liver, and skin) and organs conventionally not known to be targets of GVHD (kidneys and heart), we generated mice with organ-specific knockout of autophagy related 5 (ATG5) to specifically and exclusively inhibit autophagy in the specific organs. When compared with wild-type recipients, animals that lacked ATG5 in the gastrointestinal tract or liver showed significantly greater tissue injury and mortality, while autophagy deficiency in the skin, kidneys, or heart did not affect mortality. Treatment with the systemic autophagy inducer sirolimus only partially mitigated GVHD mortality in intestine-specific autophagy-deficient hosts. Deficiency of autophagy increased MHC class I on the target intestinal epithelial cells, resulting in greater susceptibility to damage by alloreactive T cells. Thus, autophagy is a critical cell-intrinsic protective response that promotes tissue tolerance and regulates GVHD severity.

## Abstract 2

Autophagy is a critical cell-intrinsic protective response that promotes tissue tolerance and regulates graft-versus-host disease severity.

## Introduction

Graft-versus-host disease (GVHD) is a complex pathogenic immunological consequence of allogeneic hematopoietic stem cell transplantation (allo-HSCT) that causes injury to specific organs like the gastrointestinal (GI) tract, liver, and skin and leads to significant clinical mortality and morbidity. Damage to these target tissues is incurred from the alloreactive donor T cells that recognize the disparate major or minor histocompatibility antigens on the host tissues (1–5). Strategies to prevent and alleviate GVHD focus exclusively on suppressing immune responses, which might result in infectious complications and relapse of the primary disease. While emerging data identified possible biomarkers released from damaged tissue that may be used as prognostic indicators of disease (6, 7), the precise target tissue–intrinsic mechanisms that regulate the sensitivity of the tissues to allogeneic inflammation-mediated damage are not understood (8). Thus, tissue tolerance — the tissue-intrinsic factors that protect from or amplify GVHD damage and modulate the severity of the disease without directly altering the donor T cells and systemic inflammation — remains poorly understood (8–10). We have recently demonstrated that intestinal cell metabolism is a critical regulator of intestinal epithelial cell sensitivity to T cell–mediated damage in allo/autoimmunity (11). However, whether other intestinal cell–intrinsic pathways or mechanisms also regulate immune-mediated colitis remains unknown.

Although the entire host, GVHD and non-GVHD tissues, is under continual stress from systemic inflammation and donor alloreactive cells following allo-HSCT, acute GVHD affects only specific organs, at least to the extent that they are primary causes of clinical symptoms that drive mortality and morbidity. The reasons for this limitation of organ damage might be related to tissue-intrinsic mechanisms that regulate the organs’ sensitivity to immune-mediated injury following allo-HSCT. However, it is not known whether the target cell–intrinsic pathways that play a role in mitigating tissue sensitivity to injury from pathogenic T cells are similar, or distinct between the various GVHD target organs — GI tract, liver, skin, etc. In addition, whether there is a similar or shared biological mechanism between the various target organs, and if so, whether the pathway is conserved or whether it plays a role in preventing damage of non-GVHD organs after allo-HSCT, remain unknown. Herein we addressed these questions.

Autophagy is a conserved cellular process that is highly active in cells under duress. It is a complex, coordinated mechanism regulated by specific proteins and cellular processes. Previous studies investigating the effects of autophagy on GVHD had largely focused on the role of autophagy in immune cell–specific responses (12–17), but the impact on host nonimmune cells, the epithelial cell targets of GVHD, has not been well explored. Several in vitro and in vivo studies have demonstrated that autophagy is essential for epithelial cell homeostasis (18–20). Importantly, autophagy has been shown to be induced in immune cell targets, including tumors, but whether it specifically and directly regulates the sensitivity of normal epithelial cells to in vivo alloreactive T cell–mediated nonhematopoietic GVHD target organs such as GI tract, liver, and skin has not been systematically explored.

In this study, to directly investigate the role of autophagy in the injury caused by graft-versus-host response in both target and nontarget nonhematopoietic tissues, we generated mice with tissue-specific knockout of autophagy related 5 (*Atg5*) by crossing B6-background *Atg5^fl/fl^* mice to animals containing tissue-specific *Cre* promoters (Table 1). In total, we generated knockout mice with autophagy deficiency in 5 specific tissues, 3 GVHD target tissues (gut, liver, and skin), and 2 nontarget tissues (kidney and heart). Using these strains in well-established mouse models of allo-HSCT, we found that autophagy acts as a protective mechanism to prevent tissue damage and mitigate GVHD-associated mortality only from GI and liver injury. In contrast, autophagy did not aid in protection from alloreactive T cell attack in the skin and had no role in modulation or sensitization of conventional non-GVHD target tissues such as the kidney or the heart. Mechanistic studies demonstrated that ATG5-dependent autophagy regulated the expression of major histocompatibility complex class I (MHC-I) on the surface of intestinal epithelial cells (IECs), suggesting a role for their increased sensitivity to T cell–mediated damage. Thus, our findings demonstrate that ATG5-dependent autophagy acts as a tissue-intrinsic protective mechanism in GVHD target organs but is indispensable in sensitizing non-GVHD target organs to systemic inflammation.

## Results

### Autophagy is induced in host IECs after allo-HSCT and regulates GVHD severity.

**Figure 1:**
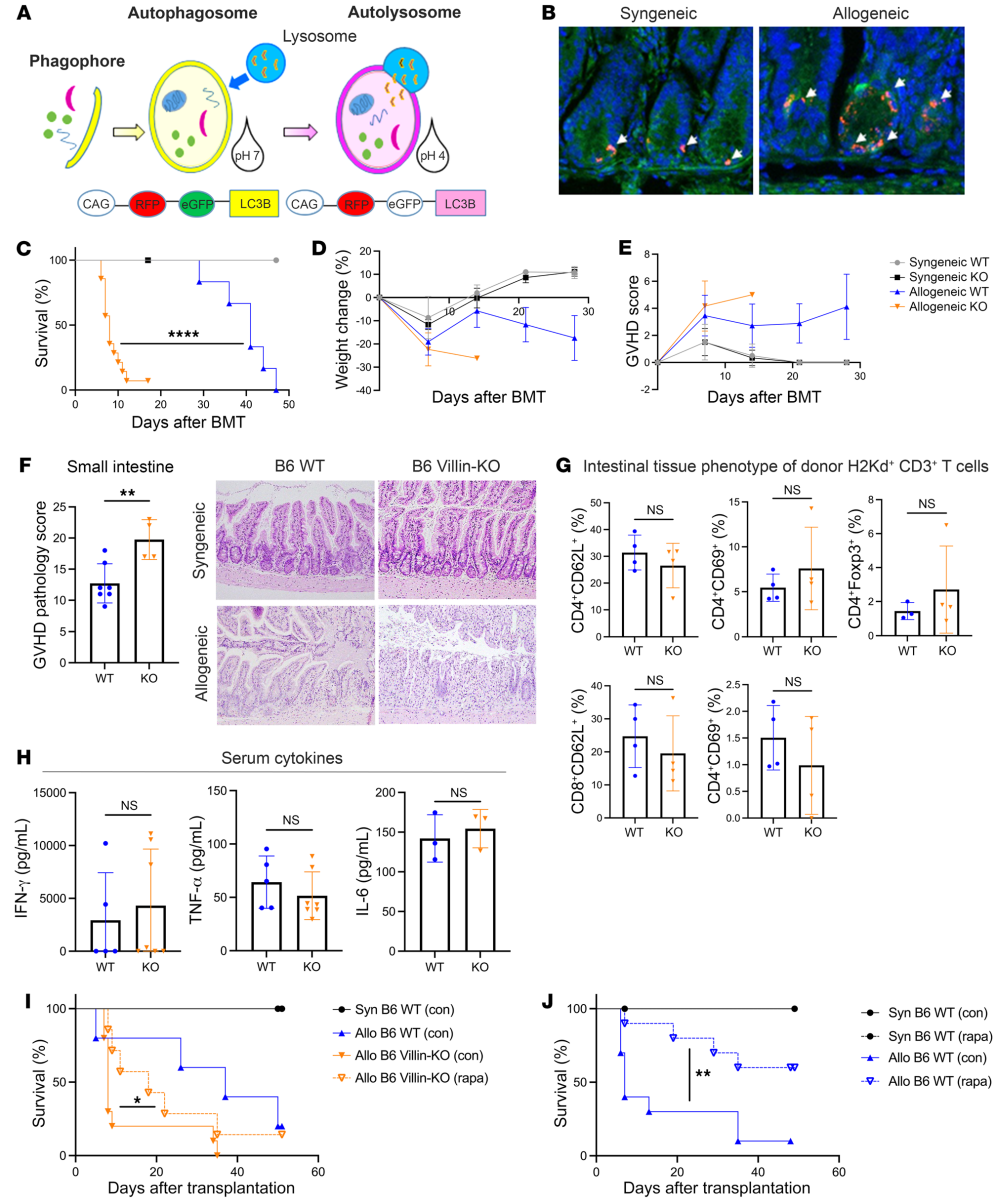
Villin-Cre^+^ *Atg5^–/–^* mice display increased mortality and a greater severity of GVHD after allo-BMT in the absence of autophagy in the gut. (**A**) Schematic of CAG-RFP-EGFP-LC3 mice with dual fluorescent expression capabilities, which distinguishes autophagosomes from autolysosomes. (**B**) Induction of autophagy on day 3 after BMT in small intestine from CAG-RFP-EGFP-LC3 recipients (original magnification, ×20). (**C**–**F**) B6 Villin-Cre^+^ *Atg5^–/–^* mice (B6 Villin-KO) and Villin-Cre^–^ *Atg5^fl/fl^* wild-type (B6 WT) littermate controls on a C57BL/6 background were used as recipients for syngeneic (syn) and MHC-mismatched allo-BMT. Mice were monitored weekly for survival (**C**), weight change (**D**), and GVHD score (**E**). (**F**) GVHD score in the small intestine on day 7 after BMT with representative micrographs (original magnification, ×20) of H&E-stained sections. (**G**) Phenotype of intestinal donor (H2K^d+^) CD3^+^ T cells at day 4 after BMT. (**H**) Concentration of serum cytokines on day 7 after BMT in B6 Villin-KO and B6 WT mice, measured by ELISA. (**I**) B6 WT and B6 Villin-KO mice received either syn- or allo-BMT; these 2 groups were split in two and treated with either sirolimus (rapa) or control (con). Survival curves after BMT. (**J**) B6 WT mice received either syn- or allo-BMT; these 2 groups were split in two and treated with either sirolimus (rapa) or control (con). Survival curves after BMT. **C**–**E** represent 2 independent experiments (Syn, *n =* 7; Allo B6 WT, *n =* 14; Allo KO, *n =* 14). **F** and **H** represent analysis on day 7 after BMT (**F**: B6 WT, *n =* 7; B6 Villin-KO, *n =* 4; **H**: B6 WT, *n =* 5; B6 Villin-KO, *n =* 7). **G** represents analysis on day 4 after BMT (B6 WT, *n =* 4; B6 Villin-KO, *n =* 4). **I** represents 2 independent experiments (syn, *n =* 4; Allo B6 WT con, *n =* 5; rapa, *n =* 10; B6 Villin KO con, *n =* 10; rapa, *n =* 7) for 50 days. **J** represents 2 independent experiments (syn B6 con, *n =* 3; syn B6 rapa, *n* = 3; allo B6 con, *n =* 10; allo B6 rapa, *n* = 10) for 50 days. Significance was determined using log-rank (Mantel-Cox) test for survival curves and unpaired *t* test for weight and GVHD score. *P < 0.05, **P < 0.01, ****P < 0.0001.

GVHD in the gut is a primary driver of morbidity and mortality after allogeneic bone marrow transplantation (allo-BMT) in both mice and humans (21–24). We therefore sought to determine whether autophagy is up- or downregulated in the host GI tract after allo-HSCT. To this end, we used B6 CAG-RFP-EGFP-LC3 reporter mice as recipients in a BALB/c (H2^d^) into B6 (H2^b^) GVHD model (25) and observed autophagic flux by confocal microscopy as described in Methods. We used these recipients because autophagosomes in these mice are tagged with a dual enhanced green fluorescent protein (EGFP)–red fluorescent protein (RFP) tag. As these autophagosomes fuse with lysosomes and pH drops, the EGFP signal is quenched, and autolysosomes can be identified by an RFP-only signal (Figure 1A). Using confocal imaging, we observed sustained autophagy in the intestinal epithelium early after HSCT, at day 3 after allo-BMT (Figure 1B). These data suggest a potential role for autophagy within GVHD target organs after allo-BMT.

We next determined whether deficiency of autophagy in the GI tract has a deleterious effect on outcomes of GVHD. To disrupt autophagy in the GI epithelium, we used C57BL/6 (B6) mice that express Cre recombinase in the intestinal epithelium under control of IEC-specific villin-1 promoter (Villin-Cre). These animals were crossed with B6 mice, in which exon 3 of the gene encoding the critical autophagy protein ATG5 is flanked by *loxP* sites (25). The resulting Villin-Cre^+^ *Atg5^–/–^* (B6 Villin-KO) mice lack autophagy specifically in the GI epithelium (Supplemental Figure 1A; supplemental material available online with this article; https://doi.org/10.1172/JCI167369DS1). To rule out potential impact of strain and microbiome, we used B6 Villin-Cre^–^ *Atg5^fl/fl^* littermate animals as B6 WT control recipients and assessed the effect of autophagy deficiency in IECs on GVHD severity following an MHC-mismatched BALB/c into B6 model of allo-HSCT. In brief, B6 Villin-KO and B6 WT mice were lethally irradiated (1,000 cGy total-body irradiation [TBI], split dose) and transplanted with 3 × 10^6^ T cells and 5 × 10^6^ T cell–depleted bone marrow cells from allogeneic BALB/c or syngeneic B6 donor mice and were followed for GVHD mortality and clinical severity, as in Methods. We observed a dramatic reduction in the survival of allogeneic B6 Villin-KO mice when compared with allogeneic B6 WT mice (Figure 1C). In contrast, both the syngeneic B6 Villin-KO and the B6 WT mice survived the entire observational period without signs of GVHD, demonstrating that deficiency of autophagy in host IECs did not impact survival from conditioning-related inflammation in the absence of alloreactivity. Notably, even though only a small number of allogeneic B6 Villin-KO mice survived for 7 days, we observed a severe drop in body weight and an increase in GVHD severity in these mice after transplant (Figure 1, D and E).

Next, to increase generalizability and reduce potential strain-related artifacts, we also tested the impact of autophagy deficiency in the host IECs in a second clinically relevant, C3H.sw into B6 model of allo-HSCT, MHC-matched but mismatched for multiple minor antigens, as described in Methods. Once again, B6 Villin-KO recipients demonstrated significantly greater mortality and GVHD severity when compared with B6 WT animals (Supplemental Figure 1, B–D).

To further confirm that the increase in mortality was a direct consequence of greater injury only to the intestinal tract in the absence of ATG5-dependent autophagy in the IECs, and not due to greater injury to other target organs from a potential increase in systemic inflammation, we performed a detailed histological assessment of GVHD target organs in a blinded manner as in Methods. We observed significantly more pronounced injury only in the small intestine of allogeneic B6 Villin-KO mice compared with B6 WT mice on day 7, as indicated by significantly higher GVHD pathology scores (Figure 1F). By contrast, we saw no significant histopathological differences in the large intestine, liver, or skin of the B6 Villin-KO and B6 WT mice after allo-HSCT (Supplemental Figure 1E). Collectively these data demonstrate that intestinal autophagy plays an important role in protecting allo-HSCT recipients from GVHD, independent of the stress induced by the conditioning regimen, or strain or degree of histocompatibility.

### Increase in GVHD severity from ATG5 deficiency in host IECs is not associated with increase in systemic inflammation.

GVHD is primarily driven by donor lymphocytes, and systemic inflammation. Therefore, it is formally possible that the IEC-protective effects of autophagy in the context of allo-HSCT might be an indirect effect due to an increase in systemic inflammation. To test this possibility, we assessed the numbers, phenotypic marker expression, and cytokine production of donor lymphocytes after allo-HSCT, temporally, just before an increase in tissue GVHD and mortality was observed, on day +3 after HSCT. No significant differences were observed in splenic donor CD4^+^ T cell numbers or CD8^+^ T cell numbers between B6 Villin-KO and B6 WT mice (Supplemental Figure 1F). Similarly, no differences in the degree of immune activation, as measured by expression of markers such as CD62L and CD69, were observed in intestinal resident donor CD4^+^ and CD8^+^ subsets, nor were any differences noted in the expansion of donor FoxP3^+^ regulatory T cells between the 2 allogeneic groups (Figure 1G). Both the B6 Villin-KO and the B6 WT allo-recipients also demonstrated similar numbers of IFN-γ–producing CD4^+^ T cells (Supplemental Figure 1G) and serum cytokine levels of proinflammatory systemic cytokines that have been implicated in GVHD severity, such as IFN-γ, TNF-α, or IL-6 (Figure 1H). It is possible that when even greater numbers of animals can be used, small differences may be observed, but these data when taken collectively suggest that deficiency of autophagy in host IECs amplifies GVHD-mediated intestinal injury and mortality without a significant change in donor T cell activation, function, and systemic inflammation.

### Allogeneic Villin-KO animals are partially rescued by treatment with sirolimus.

Autophagy in donor T cells and host antigen-presenting cells (APCs) has been shown to differentially affect GVHD (12, 17), while systemic administration of the immunosuppressive and autophagy-inducing sirolimus (rapamycin) has been shown to mitigate GVHD (26, 27). However, whether autophagy induction by sirolimus, independent of its effects on immune cells, contributes toward its regulation of GVHD is not known. Because systemic treatment with sirolimus could potentially induce autophagy in both the host and transplanted donor cells, we next evaluated whether sirolimus would protect from GVHD despite absence of autophagy induction only in the intestinal target tissues. We performed BMT using B6 WT and B6 Villin-KO animals as allo-HSCT recipients as above and in Methods, with the allogeneic hosts divided into 2 groups: one group received the mTOR inhibitor sirolimus (rapamycin), and the other received diluent control (Supplemental Figure 1H). Both allogeneic B6 Villin-KO and B6 WT animals that were treated with sirolimus displayed significantly delayed mortality when compared with respective control-treated B6 Villin-KO or B6 WT mice (Figure 1, I and J). However, sirolimus-treated allogeneic B6 WT animals showed a more pronounced rescue phenotype when compared with sirolimus-treated B6 Villin-KO animals. These data indicate that immunosuppression with sirolimus only partially rescues B6 Villin-KO mice from GVHD mortality, thus underscoring that despite the immunoregulatory effects of sirolimus on T cells (and other immune cells), its impact on induction of autophagy on host target tissues such as the GI tract is also critical for optimal benefit from sirolimus.

### Deficiency of autophagy in liver aggravates GVHD mortality.

We next explored whether autophagy is a critical tissue-protective response only in the GI tract or whether it is an important response that regulates injury in another GVHD target organ, the liver, which can also drive mortality. To this end, we generated a host that exclusively had autophagy deficiency only in the hepatic cells, by crossing B6 mice with Cre recombinase expressed under control of the Alb1 promoter to B6 *Atg5^fl/fl^* mice. The resulting mice were deficient in autophagy only within the hepatocytes of the liver (B6 Albumin-KO) and were used as recipients along with the littermate *Atg5^fl/fl^* (B6 WT) controls in the BALB/c into B6 MHC-mismatched model of GVHD as described above and in Methods.

**Figure 2:**
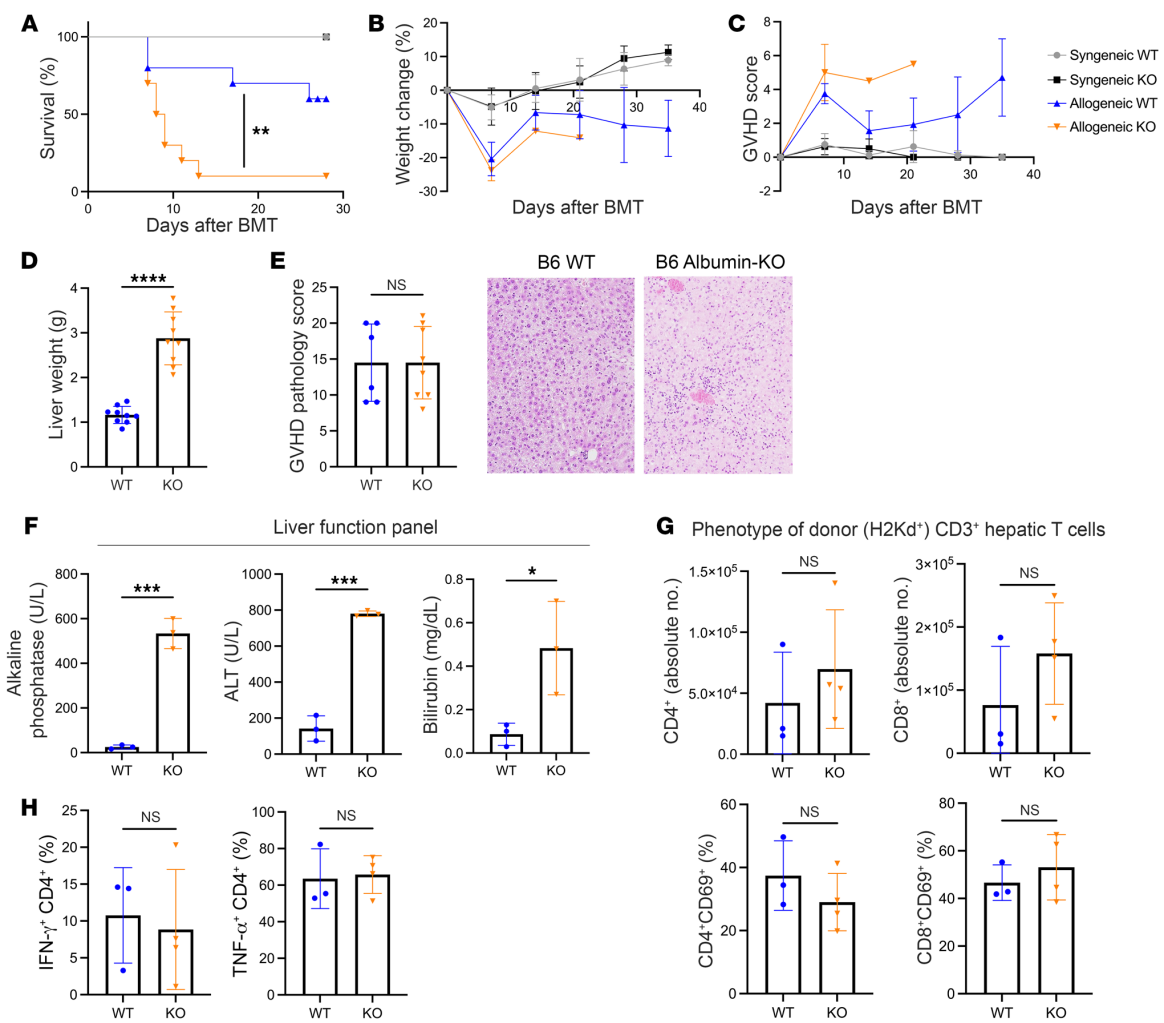
Albumin-Cre^+^ *Atg5^–/–^* mice display greater mortality after allo-BMT in the absence of autophagy in the liver. Alb-Cre^+^ *Atg5^–/–^* mice (B6 Albumin-KO) and Alb-Cre^–^ *Atg5^fl/fl^* littermate controls (B6 WT) on a C57BL/6 background were used as recipients in syn- and MHC-mismatched allo-BMT. Mice were monitored weekly for survival (**A**), weight change (**B**), and GVHD score (**C**). (**D**) Liver weights. (**E**) Liver scored for GVHD pathology at day 7 after BMT with representative micrographs (original magnification, ×20) with H&E-stained tissue sections. (**F**) Liver function test levels at day 7 after BMT of bilirubin, alkaline phosphatase, and alanine aminotransferase (ALT) in B6 Albumin-KO versus B6 WT mice. (**G**) Phenotype of donor (H2K^d+^) CD3^+^ T cells at day 4 after BMT, from liver, by flow cytometry. (**H**) Percentage of IFN-γ– and TNF-α–producing donor (H2K^d+^) CD4^+^ T cells in livers from B6 Albumin-KO and B6 WT mice at day 4 after BMT, measured by flow cytometry. In **A**–**C**, BMT data represent a combination of 2 independent experiments (Syn WT, *n =* 4; Syn Albumin-KO, *n =* 4; Allo B6 WT, *n =* 10; Allo B6 Albumin-KO, *n =* 10). **D** represents data comparing 2 groups (Allo B6 WT, *n =* 9; Allo Albumin-KO, *n =* 9). **E** represents combined data from 2 independent experiments analyzed at day 7 after BMT (Allo B6 WT, *n =* 6; Allo Albumin-KO, *n =* 8). **F** represents liver panel analysis on day 7 after allo-BMT (Allo B6 WT, *n =* 3; Allo B6 Albumin-KO, *n =* 3). **G** and **H** represent analysis on day 4 after allo-BMT (B6 WT, *n =* 3; B6 Albumin-KO, *n =* 4). Significance was determined using log-rank (Mantel-Cox) test for survival data. Significance was determined using unpaired *t* test for weight and GVHD score. *P < 0.05, **P < 0.01, ***P < 0.001, ****P < 0.0001.

Allogeneic B6 Albumin-KO animals exhibited significantly worse survival after BMT when compared with allogeneic B6 WT mice (Figure 2A). However, we observed no statistical differences in weight (Figure 2B) or GVHD scores (Figure 2C) in allogeneic B6 Albumin-KO mice compared with B6 WT controls, suggesting that the GI injury was not significantly different between the groups. By contrast, after HSCT, the livers from the B6 Albumin-KO recipient animals were significantly larger (Figure 2D). Significant histopathological differences were found in the B6 Albumin-KO compared with the B6 WT controls. The allogeneic and syngeneic B6 Albumin-KO recipients demonstrated significant liver ductular reaction, which is characterized by the proliferation of reactive bile ducts, compared with allogeneic and syngeneic B6 WT recipients, respectively (Figure 2E). Furthermore, when compared with allogeneic B6 WT recipients, the allogeneic B6 Albumin-KO recipients demonstrated significantly greater severity of clinical hepatic damage with increased levels of liver function tests such as bilirubin, alkaline phosphatase, and alanine aminotransferase (Figure 2F). By contrast, both WT and B6 Albumin-KO animals demonstrated similar liver function tests in the naive and syngeneic groups (Supplemental Figure 2, A and B). By contrast, the other GVHD target organs, such as the GI tract or skin, did not demonstrate significant histopathological differences in B6 Albumin-KO animals compared with B6 WT animals after allo-HSCT (Supplemental Figure 2C).

### Deficiency of hepatic autophagy does not alter donor lymphocyte engraftment or function.

To rule out any potential impact on immune activation within the target tissues, we next analyzed liver-resident lymphocytes at day 4 after BMT and detected no differences in either the numbers of donor CD4^+^ and CD8^+^ T cells or the levels of CD69^+^ subsets of donor CD4^+^ or CD8^+^ T cells in B6 WT and B6 Albumin-KO mice (Figure 2G). In addition, we observed similar numbers of CD4^+^IFN-γ^+^ and CD4^+^TNF-α^+^ hepatic-resident lymphocytes in both groups (Figure 2H). We next sought to assess a potential indirect role or an impact of loss of host hepatic autophagy on the donor T cells that might contribute to GVHD mortality. We performed flow cytometric analysis of day 3 splenic donor lymphocytes to explore impact on systemic donor T cell responses. The total numbers of donor CD4^+^ and CD8^+^ T cells, as well as the levels of donor and CD69^+^ CD4^+^ and CD8^+^ T cell subsets, were not significantly different in allogeneic B6 Albumin-KO and B6 WT mice (Supplemental Figure 2D). Consistent with these findings, we also detected no differences in the levels of serum cytokines IFN-γ, TNF-α, and IL-6 in allogeneic B6 Albumin-KO and B6 WT mice (Supplemental Figure 2E). These data demonstrate that deficiency of autophagy in the host liver only is a critical GVHD protective mechanism in hepatocytes after allo-HSCT.

### Cutaneous autophagy deficiency does not alter survival after allo-BMT.

**Figure 3:**
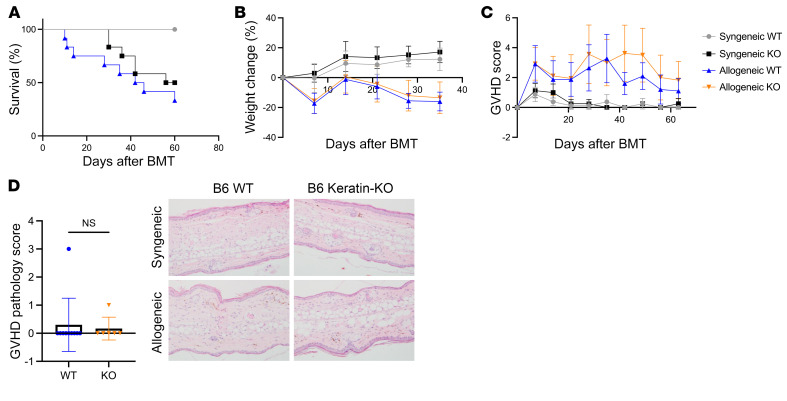
Keratin-Cre^+^ *Atg5^–/–^* mice display no phenotype after allo-BMT in the absence of autophagy in skin. Ker-Cre^+^ *Atg5^–/–^* mice (B6 Keratin-KO) and Ker-Cre^–^ *Atg5^fl/fl^* littermate controls (B6 WT) on a C57BL/6 background were used as recipients for MHC-mismatched allo-BMT. Mice were monitored weekly for survival (**A**), change in weight (**B**), and progression of GVHD (**C**) in B6 Keratin-KO and B6 WT mice after BMT. (**D**) GVHD pathology scores and representative micrographs (original magnification, ×20) with H&E-stained sections of skin, taken from the ear, at day +75 after BMT. In **A**–**C**, BMT data represent a combination of 2 independent experiments (Syn B6 WT, *n =* 4; Syn B6 Keratin-KO, *n =* 4; Allo B6 WT, *n =* 12; Allo B6 Keratin-KO, *n =* 12). **D** represents a combination of 2 independent experiments (Allo B6 WT, *n =* 10; Allo B6 Keratin-KO, *n =* 6). Significance was determined using log-rank (Mantel-Cox) test for survival data. Significance was determined using unpaired *t* test for weight and GVHD score.

Cutaneous GVHD is common but is seldom the primary driver of mortality in the clinic or in most MHC-mismatched models of murine GVHD (28, 29). Therefore, we next explored whether autophagy in the skin regulated GVHD severity and mortality as observed in 2 other acute GVHD target organs, the gut and liver. We generated B6 animals with skin-specific ATG5 deficiency by using animals with a cutaneous-organ-specific, keratin 14–specific (Krt14) Cre promoter and crossing them with *Atg5^fl/fl^* mice. The resulting mice were thus deficient in autophagy within the keratinocytes of the skin (B6 Keratin-KO). These animals and the *Atg5^fl/fl^* control mice (B6 WT) then were tested in the same, BALB/c into B6 MHC-mismatched model of GVHD. To increase GVHD severity and maximize the involvement of cutaneous GVHD, we used a single 1,000 cGy dose of TBI to condition recipient mice, rather than the split doses used in prior experiments. In contrast to the results in other GVHD target tissues, intestines and liver, we observed similar survival rate, weight change, and GVHD score in the B6 Keratin-KO and B6 WT mice (Figure 3, A–C). Furthermore, we observed no differences in histological GVHD severity in the skin at a much later time point (day +75) after transplant (Figure 3D). To rule out site and sampling artifact, skin samples were harvested from additional (alternate) sites such as the dorsal region of the recipient animals. The skin from these additional sites also did not demonstrate significant difference in GVHD pathology (Supplemental Figure 3A). Furthermore, no significant differences in GVHD histopathology were observed in the other target organs, namely the GI tract or the liver, between the B6 Keratin-KO and the B6 WT allogeneic animals (Supplemental Figure 3B). These data suggest that in contrast to cytoprotective effects in GI and liver from GVHD, autophagy does not play a major protective role against skin GVHD.

### Deficiency of ATG5 in the kidney and heart does not alter outcomes after allo-BMT.

Acute GVHD typically affects GI, liver, and skin tissues even though the MHC-disparate antigens are expressed systemically in all the host tissues. Specifically, heart and kidneys are not immune-privileged sites, are known targets of autoimmunity and are rejected, but have not been implicated in acute GVHD. Based on our initial finding that autophagy is induced in GVHD target organs, we next examined the effects of autophagy loss in nontraditional GVHD target organs, including the kidney and heart, and determined whether deficiency of autophagy might induce graft-versus-host damage to these tissues. We again crossed mice with kidney and heart organ-specific (Nphs and Myh6) Cre recombinase drivers to *Atg5^fl/fl^* mice and used these animals as recipients in allogeneic BMT following a single dose of 1,000 cGy TBI to condition recipients as in Methods.

**Figure 4:**
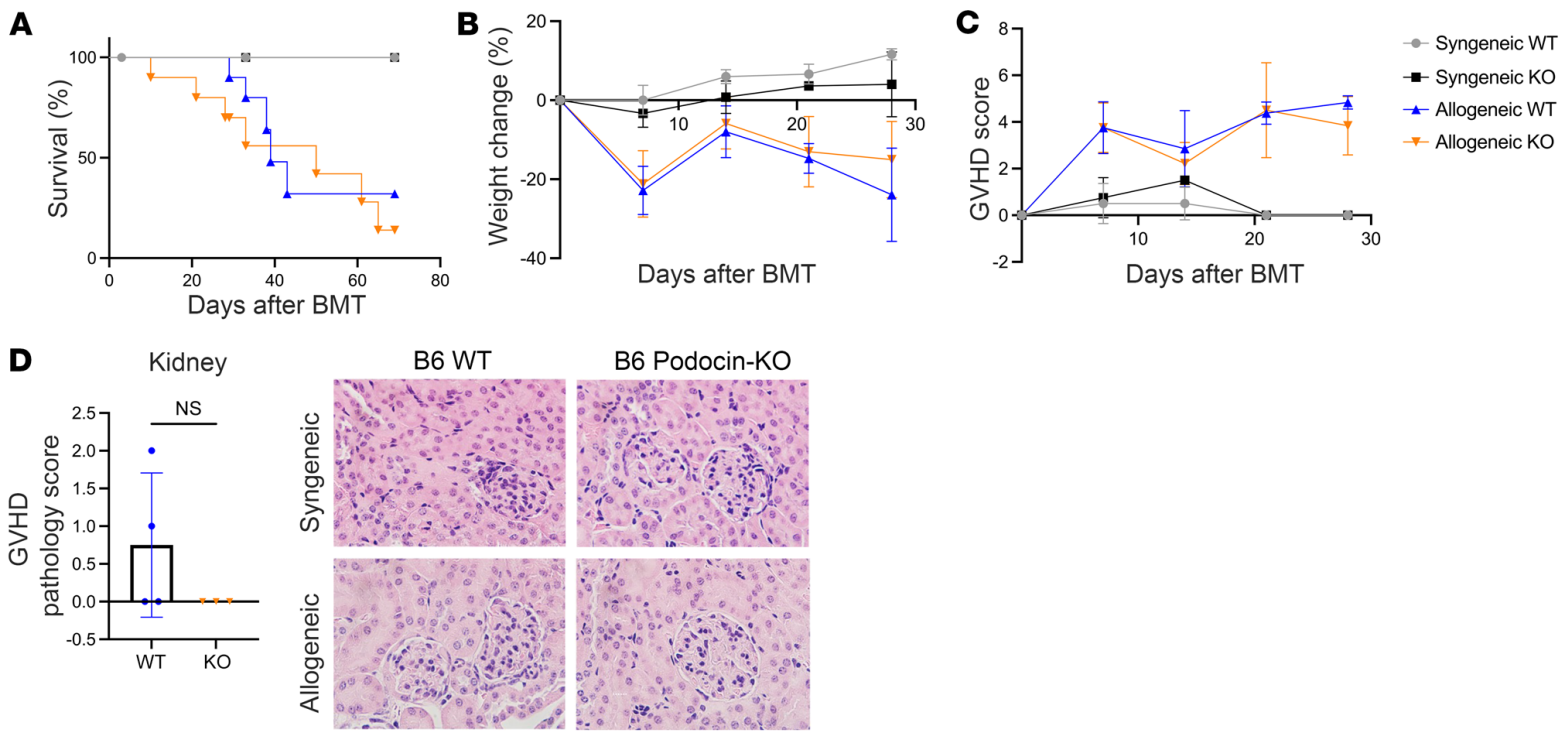
Nphs2-Cre^+^ *Atg5^–/–^* mice are similar to WT after allo-BMT in the absence of autophagy in kidneys. Nphs2-Cre^+^ *Atg5^–/–^* mice (B6 Podocin-KO) and Nphs2-Cre^–^ *Atg5^fl/fl^* littermate controls (B6 WT) on a C57BL/6 background were used as recipients for MHC-mismatched allo-BMT. Mice were monitored weekly for survival (**A**), change in weight (**B**), and progression of GVHD (**C**) in B6 Podocin-KO and B6 WT mice after BMT. (**D**) GVHD pathology scores and representative micrographs (original magnification, ×60) with H&E-stained sections of kidneys from B6 Podocin-KO and B6 WT mice at 4–8 weeks after BMT. In **A**–**C**, BMT data represent a combination of 2 independent experiments (Syn B6 WT, *n =* 3; Syn B6 Podocin-KO, *n =* 4; Allo B6 WT, *n =* 10; Allo Podocin-KO, *n =* 10). **D** represents remaining mice after 4- to 8-week survival studies comparing 2 groups (Allo B6 WT, *n =* 4; Allo Podocin-KO, *n =* 3). Significance was determined using log-rank (Mantel-Cox) test for survival data. Significance was determined using unpaired *t* test for weight and GVHD score.

To assess the role of autophagy within the kidneys in our GVHD model, we used Nphs2-Cre mice, in which Cre expression is driven by a podocyte-specific promoter (30). When these animals are crossed with *Atg5^fl/fl^* mice, the offspring lack autophagy specifically within podocytes of the kidney glomeruli (B6 Podocin-KO). After BMT, we detected no significant differences in survival, weight change, or GVHD scores between allogeneic B6 Podocin-KO and B6 WT mice (Figure 4, A–C). In addition, histological GVHD severity in the kidney and in other organs on day 7 after BMT was similar in both allogeneic groups (Figure 4D and Supplemental Figure 4A). Even when animals were observed at a later time point post-BMT, no differences in GVHD severity were observed between the B6 Podocin-KO and B6 WT animals (Supplemental Figure 4B).

To assess the role of autophagy in the heart in our GVHD model, we used Myh6-Cre mice, in which Cre expression is driven by a promoter specific to cardiac myocytes (31). When Myh6-Cre mice are crossed with *Atg5^fl/fl^* mice, the offspring are deficient in autophagy throughout the heart (Myosin-KO). After BMT, we observed that autophagy was increased in myocytes when we used B6 CAG-RFP-EGFP-LC3 reporter mice as recipients as seen in Figure 1A (Supplemental Figure 4C). Post-BMT allogeneic B6 Myosin-KO and B6 WT mice displayed similar mortality rates, weight change, and GVHD scores (Supplemental Figure 4, D–F). These results suggest that autophagy loss is not sufficient to induce GVHD in classical nontarget organs after allogeneic BMT and show that tissue-intrinsic protective mechanisms against alloreactivity, such as autophagy, are tissue specific and distinct in different tissues.

### Mechanism of autophagy-induced protective responses to GVHD in target organs.

**Figure 5:**
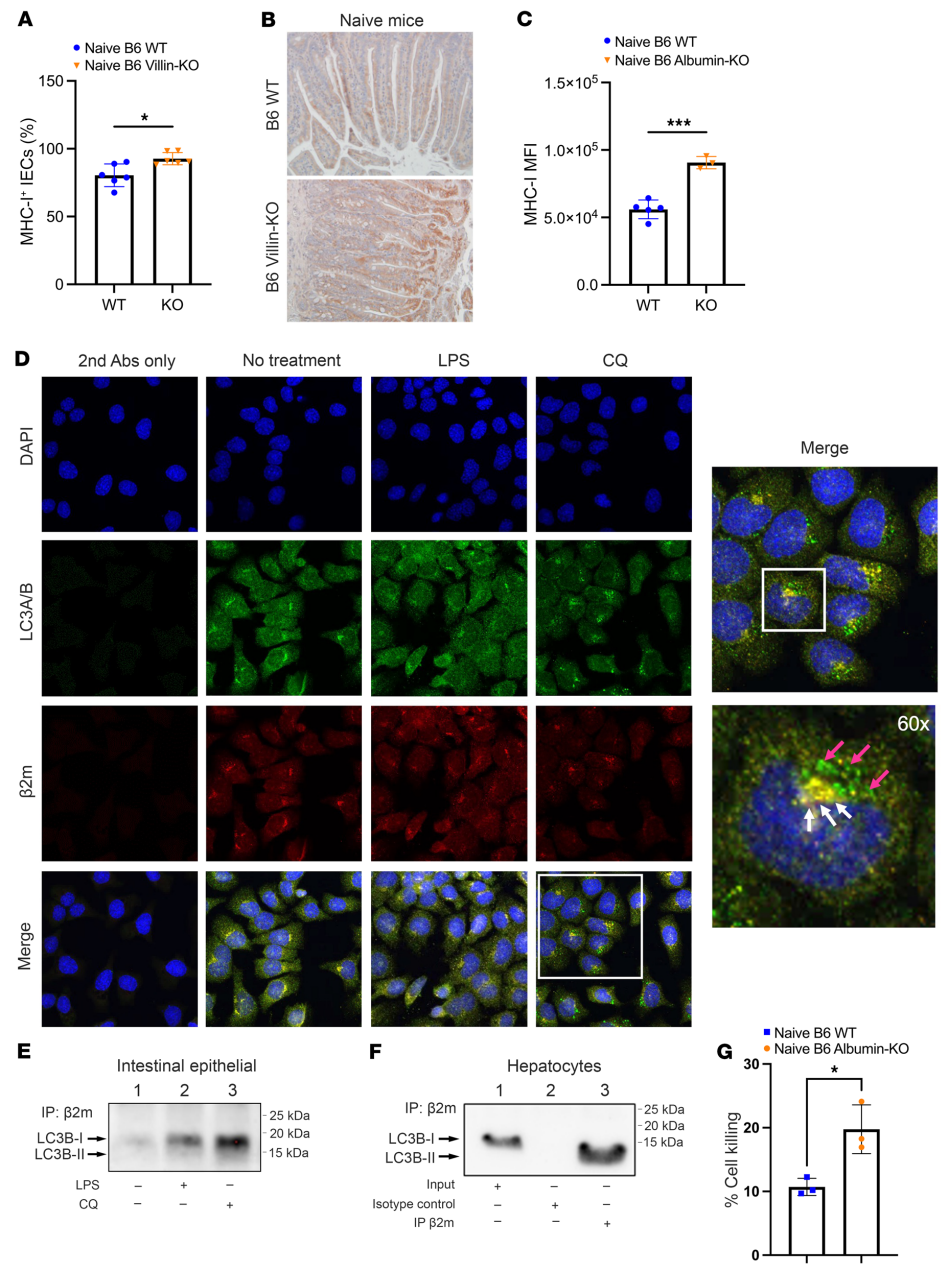
IECs from Villin-Cre^+^ *Atg5^–/–^* mice show increased levels of MHC-I compared with WT cells. (**A**) Total number of MHC-I^+^ IECs isolated from the small intestine of naive B6 Villin-KO mice and B6 WT littermates. Cells were stained with H2K^b^ antibodies and analyzed by flow cytometry. (**B**) Representative micrographs (original magnification, ×20) with immunohistochemical staining for MHC-I (β2M) on small intestine tissue from naive B6 Villin-KO and B6 WT mice. (**C**) Primary mouse hepatocytes were stained with H2K^b^ antibody and analyzed by flow cytometry. (**D**) Complete immunofluorescence panel of single-color and merged images from PCEC lines treated with LPS or hydroxychloroquine (CQ). Representative micrographs (original magnification, ×20) were stained with antibodies for MHC-I (β2M) and LC3 (LC3A/B) and analyzed via confocal microscopy. Perinuclear yellow colocalization (white arrows) can be observed, as well as accumulation of green cytoplasmic autophagosomes (magenta arrows) in CQ-treated cells. (**E**) Lysates from PCEC lines treated with LPS or CQ and control-treated cells were subjected to immunoprecipitation (IP) with MHC-I (β2M) antibody and analyzed by Western blot with LC3A/B antibody. (**F**) Lysates from untreated primary mouse hepatocytes were subjected to IP with MHC-I (β2M) antibody and analyzed by Western blot with LC3B antibody. (**G**) Primary mouse hepatocytes from B6 WT or B6 Albumin-KO mice were cocultured with activated BALB/c T cells, and cell death was measured after 4 hours. **A** represents data from naive mice (B6 WT, *n =* 6; B6 Villin-KO, *n =* 6). **C** represents data from naive mice (B6 WT, *n =* 5; B6 Albumin-KO, *n =* 3). **E** and **F** represent analysis from 1 experimental run. **G** represents analysis from 1 experimental run. Significance was determined using unpaired *t* test. *P < 0.05, ***P < 0.001.

We next analyzed the putative mechanisms underlying the protective role of autophagy against GVHD in the intestine after allogeneic HSCT. Because autophagy has been shown to regulate expression of MHC in professional APCs (32), we hypothesized that it may also regulate the expression of MHC-I even at baseline homeostasis, only in certain epithelial cells such as the IECs, where it had a protective effect against tissue injury. To test the hypothesis, we isolated small-intestinal CD326^+^ IECs in the naive state from B6 Villin-KO and B6 WT animals and assessed for MHC-I with flow cytometry and immunohistochemical staining. We found that naive B6 Villin-KO IECs showed significantly increased expression of MHC-I in small intestine (Figure 5, A and B). To confirm that these data were consistent in other GVHD target tissues, we isolated primary hepatocytes from naive mice via collagenase perfusion and Percoll gradient separation as previously described, with minor modifications (33). The B6 Albumin-KO hepatocytes showed significantly increased expression of MHC-I by flow cytometry (Figure 5C) and by Western blot (Supplemental Figure 5B), demonstrating similar effects in both IECs and hepatocytes.

Next, to demonstrate that autophagy was directly responsible for regulation of MHC-I expression on the IEC surface, we analyzed whether autophagosomes directly catalyzed the cell surface MHC-I. We treated intestinal epithelial cell lines, primary colonic epithelial cells (PCECs), either with control diluent or LPS or with hydroxychloroquine (CQ) to inhibit autophagy and analyzed them by confocal microscopy for colocalization of autophagosomes and MHC-I. We observed a clear treatment-dependent increase in levels of colocalization between MHC-I (β_2_-microglobulin [β2M], red) and the autophagosomal membrane protein LC3A/B (green). Perinuclear yellow colocalization (white arrows) can be observed, as well as accumulation of green cytoplasmic autophagosomes (magenta arrows) in CQ-treated cells (Figure 5D).

Next, analysis of whole-cell lysate samples by Western blot further demonstrated that CQ-treated PCECs produced higher levels of LC3 than untreated or LPS-treated cells (Supplemental Figure 5A). Immunoprecipitation experiments were performed with lysates from PCECs treated as above with β2M (class I) antibody, followed by Western blot with LC3A/B antibody. A significantly greater amount of LC3 protein was pulled down by β2M in CQ-treated cells, confirming a direct interaction between MHC-I and autophagosomes (Figure 5E) in the intestinal cells. We next explored whether this mechanism also contributed to the differences in hepatic GVHD. To this end, we analyzed primary mouse hepatocytes that were harvested from naive animals. Similarly to the PCECs, hepatocyte cell lysates were immunoprecipitated with β2M (class I) antibody, followed by Western blot with LC3B antibody, and showed a strong interaction between β2M and LC3B (Figure 5F). This suggests that autophagy protects GVHD target tissues, at least in part, via downregulation of MHC-I. As elevated levels of MHC-I would yield increased surface targets for allogeneic T cells, these findings provide a putative mechanism for the detrimental outcomes observed in allogeneic B6 Villin-KO and B6 Albumin-KO mice after BMT. To determine whether increased MHC-I expression on the surface of B6 Albumin-KO hepatocytes contributes to increased cell death, we performed a cytotoxic T lymphocyte killing assay. Briefly, we cocultured activated effector allogeneic BALB/c T cells with target primary mouse hepatocytes from either B6 WT or B6 Albumin-KO animals and measured cell death by chromium-51 release. B6 Albumin-KO hepatocytes showed significantly greater cell death when compared with B6 WT controls (Figure 5G). Next, to further demonstrate that this mechanism is unique only to these two GVHD target organs and is likely the reason for not observing an increase in nontarget organ toxicity, we also examined primary mouse cardiac and kidney cells for interaction between MHC-I (β2M) and LC3. In contrast to intestinal and hepatic cells, both cardiac and kidney cells did not demonstrate a direct interaction, suggesting that autophagy regulates MHC-I in a tissue-specific manner (Supplemental Figure 5, C and D).

Collectively, results from this study suggest that autophagy is a tissue-intrinsic protective response to alloimmune damage after allo-HSCT in critical GVHD target organs like GI tract and liver, but it does not play a similar role in skin or the nontarget organs. Furthermore, this protective effect is mediated, at least in part, by downregulation of MHC-I on the surface of cells within GVHD target tissues.

## Discussion

Many studies have investigated how systemic immune responses contribute to the regulation of acute GVHD severity, and recent investigations have also started to explore the function of tissue-resident immune cells in disease severity. However, seldom have studies focused specifically and exclusively on the mechanisms intrinsic to target tissues or cells that regulate their resilience in the context of a pathogenic attack by alloreactive T cells (34–37). Tissue tolerance has previously been explored by our laboratory as a model for understanding the tissue-specific programs that contribute to target-tissue resilience, repair, and regeneration, and mitigate severity of acute GVHD, without altering the load or function of alloreactive immune cells (8). Autophagy has been shown to play an active and protective role in numerous diseases, including inflammatory bowel disease, Crohn’s disease, and multiple types of cancers (38, 39). In this study, we explored whether a well-characterized protective cellular process, autophagy, exclusively in the specific target tissues may play a protective role in GVHD pathology. We found that autophagy was protective in mitigating tissue-specific damage and mortality from GVHD only in GI tract and liver and had no impact on skin or on susceptibility of nontarget organs to pathogenic alloreactive T cell–mediated damage. Mechanistically, it is in part dependent on the regulation of MHC-I on the target cells. However, autophagy regulates several processes, including the turnover of many key proteins. Thus, is it possible that additional mechanisms may account for the protective effects, which will need to be explored in future studies. For instance, it was recently shown that in the absence of autophagy, goblet cells within the intestine are depleted, or the ability of intestinal stem cells may be altered and exacerbate intestinal damage (1, 7, 19, 40–43).

Autophagy is induced in target tissues by the inflammation caused, in the context of allo-HSCT, by conditioning and alloreactive T cells (44). It has been shown to affect donor HSCs and donor T cells and host APCs with variable effects on GVHD severity (12, 13, 17, 45). Specifically, reduction in autophagy in hematopoietic APCs exacerbated GVHD, while deficiency of autophagy in donor T cells attenuated GVHD severity. Clinical use of autophagy inducers and inhibitors impacts GVHD in a variable manner as well (26, 46–48). Thus, the role of autophagy in GVHD as a whole remains ambiguous and poorly understood. Herein we provide insight into the role of autophagy with a focus on all of the host nonimmune, epithelial target tissues. Our data demonstrate that autophagy is a protective response of the target tissues against GVHD in intestines and liver. Intriguingly, it had no measurable impact on disease severity in skin and in other host organs like heart and kidneys. These data highlight the notion that not all tissues adopt autophagy or other similar cell-intrinsic protective or stress response programs to withstand inflammation. These observations also raise additional questions: does the severity or type of inflammation or pathogenic T cell attack elicit distinct protective programs in the same cells and tissues? These questions and specific mechanisms will need to be addressed in carefully designed future studies. Nonetheless, our data clearly demonstrate that autophagy is a critical regulator of GVHD mortality even when restricted to a single target organ such as the GI tract or liver. Notably, we further observed only partial rescue of the ATG5 Villin-KO hosts treated with the mTOR inhibitor sirolimus, which induced autophagy, as compared with untreated ATG5 Villin-KO mice. Sirolimus use mitigates experimental and clinical GVHD (49–51), albeit not completely. This might be because of its immunosuppression that is linked, or unlinked, to autophagy induction on T cells and APCs (49, 52). The direct impact of autophagy induction in its immunomodulating effects in the context of GVHD remains to be explored. However, the observation that only a partial rescue is observed in ATG5 Villin-KO animals treated with sirolimus demonstrates clearly that at least part of GVHD protective effects is dependent on its ability to induce autophagy in target organs like the GI tract.

Previous reports have demonstrated that deficiency of autophagy increased the susceptibility of intestinal organoids ex vivo to cell death from necroptosis by alloreactive T cells and from GVHD (18, 20). Both the studies used ATG16LI^IEC^ to disrupt autophagy in IECs and reported a timeline of mortality after BMT similar to that seen in our study with ATG5 Villin-KO animals (18). Taken together, these studies demonstrate that the increase in GVHD mortality is a direct consequence of loss of autophagy as a protective response to an alloreactive T cell–mediated attack on all villin^+^ IECs, thus lending direct evidence to the notion of tissue tolerance after sterile inflammation (8, 10). However, our study has limitations. First, it does not address the impact of autophagy in various intestinal cell subsets, particularly those in the intestinal crypts, including the intestinal stem cells that are critical targets of GI GVHD. This remains a limitation that will need to be addressed by targeting of autophagy in specific GI cell subsets, including the intestinal stem cells, Paneth cells, and goblet cells, all of which are targets of alloreactive T cells and have been shown to regulate GI GVHD (53). Second, the microbiome is a critical regulator of GVHD; it also regulates IEC-specific autophagy and impacts GI infections (54). Therefore, it would be important for future studies to explore how the microbiome regulates target tissue–specific autophagy and whether interrupting those pathways may regulate GVHD outcomes.

Our study extends the protective role of autophagy to second GVHD target tissues that can also drive mortality from GVHD, namely liver. To our knowledge, this is the first report of a liver tissue-specific pathway being shown to directly contribute to GVHD. It must, however, be noted that the liver pathology characterized by ductular reaction is not unique to GVHD but is also recognized in biliary disorders as well as in nonalcoholic fatty liver disease (55). Liver injury can result from many different stressors, and abrogation of autophagy (56) has been linked to various pathogenic hepatic processes like nonalcoholic steatohepatitis, liver fibrosis, tumorigenesis, and insulin resistance (57, 58) and models of acute liver injury (59, 60). When taken collectively with our data, these results suggest that autophagy plays a cytoprotective role in recovery from several types of liver damage and is also germane to recovery from and/or tolerance to hepatic GVHD. The fact that the syngeneic ATG5 Albumin-KO animals recovered after BMT despite showing some histopathological changes suggests that the critical role played by hepatic autophagy might be partly related to the degree of inflammation or the severity of the damage.

Skin GVHD causes morbidity and, rarely, mortality in patients. Moreover, in C57BL/6 mice, the skin has been shown to be a GVHD target organ, albeit not one that drives mortality (29). Thus, although autophagy has been shown to play an important role in wound healing (61), it is perhaps not surprising that we observed no differences in mortality or weight between ATG5 Keratin-KO and WT recipients. Nonetheless, it must be noted that our study is limited to one model system of skin GVHD. Thus, future studies will need to address whether autophagy plays a tissue-specific cytoprotective role in regulation of acute or chronic skin GVHD in additional model systems of GVHD (29).

One of the central enigmas of the clinical presentation of acute GVHD is why relatively few organs are affected even though the antigens driving alloreactive T cells are present in the entire host. To attempt to answer this question, we hypothesized that non-GVHD target organs might demonstrate significantly greater autophagy and thus are protected from the damage caused by alloreactive T cells. To test this hypothesis, we used animals that are specifically, and only, deficient in autophagy in either the kidney or the heart (traditionally not acute GVHD targets) as recipients in allo-HSCT (62–67). Furthermore, in both of these organs, autophagy has been demonstrated to play a cytoprotective role and maintain homeostasis (68–70). However, contrary to our hypothesis, neither in the kidney nor in the heart did deficiency of autophagy increase GVHD mortality or severity after allo-BMT, suggesting that it does not play a protective role against GVHD in these nontarget tissues. Thus, tissue-intrinsic autophagy regulates GVHD damage in the GI tract and liver but has no role in skin or in other atypical target tissues for clinical GVHD. Future studies will need to address whether any other cell-intrinsic responses might be responsible for target organ selectivity in acute GVHD (8).

Our data suggest that the mechanisms of the protective effects against alloreactive cytotoxic T cells of cellular autophagy in IECs appear to be due to its role in regulation of MHC-I expression on the surface of the target cells. Both flow cytometry and immunohistochemistry experiments demonstrated greater MHC-I expression in IECs from ATG5 Villin-KO animals compared with WT animals. The increase in MHC-I expression may lead to a greater number of targets for cytotoxic T cells and thus greater damage to IECs. Furthermore, our data confirm colocalization of MHC-I and autophagosomes in PCECs and hepatocytes, suggesting that autophagy directly regulates class I expression on the cell surface. These results are consistent with and extend the reports demonstrating increased surface levels of MHC-I on autophagy-deficient dendritic cells (32, 71, 72), and that autophagy plays a role in antigen cross-presentation (73, 74) to IECs. In addition, MHC-II regulation has also been associated with autophagy (75). However, we did not find a significant difference in the expression of MHC-II between the WT and ATG5 Villin-KO animals. It remains to be determined whether expression of class II is regulated in other target tissues and whether this plays a protective role against CD4^+^ T cell–mediated GVHD. Nonetheless, the process of MHC-I autophagy-driven degradation was also shown to be exploited by certain tumor cells to avoid antitumor T cell responses (76). However, it is important to consider that, given the broad and, likely, tissue- and context-specific role of autophagy in regulation of cellular proteins and tissue homeostasis, regulation of other proteins besides or independent of MHC-I may contribute to GVHD damage. This will need to be assessed in future studies with unbiased integrative analysis across several models and tissues. Our results suggest that clinically, in severe immunosuppressive-resistant GVHD, such as in steroid-refractory GVHD, strategies that enhance target-tissue autophagy and promote tissue tolerance may serve as critical adjuncts for mitigating GVHD mortality. It is likely that autophagy inducers such as sirolimus or development of novel therapeutics that exclusively induce autophagy without other immunosuppressive properties may have a role in steroid-refractory GVHD. Similarly, our data suggest that using agents that are immunosuppressive and also inhibit autophagy, such as CQ, may have detrimental effects. Future studies will need to address whether this mechanism is germane to graft-versus-tumor responses observed after allo-HSCT and, if so, in what hematological malignancies. Thus, autophagy-dependent protective responses to T cells could be adapted by distinct tissues (77).

In conclusion, results from this study show that autophagy plays a protective role in GVHD target tissues, whereas in contrast, loss of autophagy has no effect on either the skin or non-GVHD target tissues, such as the kidney and heart, after allo-HSCT. Moreover, the observation that the protective effects of target-tissue autophagy are exclusive to allo-BMT recipients suggests they are independent of conditioning toxicity. Thus, autophagy can regulate tissue resilience against GVHD in GI and liver and improves mortality by promoting their tolerance of alloreactive T cell–mediated damage even in the absence of immunosuppression.

## Methods

### Sex as a biological variable.

Our study examined male and female animals, and similar findings are reported for both sexes.

### Reagents.

Carboxymethylcellulose sodium (CMC; Sigma-Aldrich) was prepared in sterile water. Sirolimus (rapamycin) (MilliporeSigma) was reconstituted in DMSO at a concentration of 100 mM; further dilutions were made in a 0.2% CMC solution. CQ (MilliporeSigma) was reconstituted in PBS at a concentration of 100 mM, and further dilutions were made in Roswell Park Memorial Institute (RPMI) medium. Lipopolysaccharide (LPS; MilliporeSigma) was reconstituted in sterile water at 1 mg/mL, and further dilutions were made in RPMI medium.

### Mice.

B6 CAG-RFP-EGFP-LC3 reporter mice, C57BL/6 (B6, H-K2^b^) mice, and BALB/c (H-K2^d^) mice were purchased from The Jackson Laboratory and Charles River Laboratories. Previously described B6-background *Atg5^fl/fl^* mice (B6.129S-ATG5<tm1Myok>, RBRC02975, Riken RBC) (15, 78) were bred to multiple-tissue-specific transgenic Cre mice (Table 1).

### BMT.

BMT was performed as previously described (79, 80). Briefly, splenic T cells from donor mice were enriched using the Pan T Cell Isolation Kit II and manual MACS separation with LS columns (Miltenyi Biotec Inc.). Bone marrow was depleted of T cells by manual MACS separation with CD90.2 microbeads (Miltenyi Biotec Inc.). For BMT experiments, we used the well-established MHC-mismatched BALB/c→B6 BMT model (81), in which BALB/c animals act as donors and B6 mice are recipients. All *Atg5*-knockout strains were used as recipients and were irradiated with either a split dose (villin, albumin) or a single dose (keratin, podocin, and myosin) of 10 Gy (^137^Cs source) on day –1 relative to BMT. C57BL/6 mice were irradiated with a single dose of 10 Gy on day –1 relative to BMT. Animals then received 3.0 × 10^6^ CD90.2^+^ T cells along with 5 × 10^6^ T cell–depleted bone marrow (TCD-BM) cells from either syngeneic (B6) or allogeneic (BALB/c) donors on day 0.

### Systemic and histopathological analysis of GVHD.

Survival of animals that received transplanted cells was monitored daily, and the degree of clinical GVHD was assessed weekly, as described previously (82). Histopathological analysis of GVHD target and nontarget organs was performed, as described, using a semiquantitative scoring system that was implemented in a blinded manner by a single pathologist

### Isolation of intestinal epithelial cells and intraepithelial cells.

Luminal contents from dissected small and large intestines were flushed with CMF buffer (Ca^2+^/Mg^2+^-Free Hanks balanced salt solution, Thermo Fisher Scientific), supplemented with 25 mM sodium bicarbonate (Sigma-Aldrich) and 2% FBS (Gemini Bio Products). Intestines were then minced into 5 mm pieces, washed with CMF buffer 4 times, transferred to CMF with 5 mM EDTA (Lonza), and incubated at 37°C for 40 minutes (with shaking of tubes every 10 minutes). Supernatants containing intestinal epithelial cells (IECs) were transferred through 100 μm cell filters, followed by incubation on ice for 10 minutes to allow sedimentation. Supernatants were then transferred through a final 75 μm cell filter.

### Rapamycin-treated animals.

Villin-Cre^+^ *Atg5^–/–^* (B6 Villin-KO), Villin-Cre^–^ *Atg5^fl/fl^* littermate control (B6 WT), and C57BL/6 mice were used as BMT recipients, as described above. After BMT on day 0, recipients started daily intraperitoneal injections of either diluent control (CMC) or sirolimus (2rapamycin; Cayman Chemical) at a dose of 4.5 mg/kg/mouse for 14 days, after which dosing shifted to 3 d/wk for the remainder of the study. GVHD scoring was recorded weekly (50).

### FACS analysis.

FACS analysis to assess lymphocytic phenotypes was performed using the following antibodies: anti–mouse CD3, CD4, CD8, CD62L, CD69, FoxP3, and IFN-γ (BioLegend). For staining of MHC-I, purified IECs were harvested after stimulation, washed twice with FACS wash buffer (0.2% BSA in PBS), and fixed with 1× BD FACS Lysing Solution (BD Biosciences). Cells were then permeabilized for 10 minutes at 4°C with 1× Permeabilization Buffer (eBioscience) in the presence of 1:400 rat anti–mouse FcR monoclonal antibody 2.4G2 (BD Biosciences) to block nonspecific FcR binding of labeled antibodies. After blocking, cells were incubated with primary rabbit MHC-I antibody directly conjugated to PE (H2K^b^-PE, BioLegend) diluted 1:200 in Permeabilization Buffer for 30 minutes at 4°C, washed once, and then incubated for 30 minutes at 4°C in Permeabilization Buffer at the same concentration. Stained cells were then resuspended in FACS wash buffer and analyzed using the Attune NXT cytometer (Thermo Fisher Scientific). For MHC-I staining in primary hepatocytes, cells were harvested and washed twice with FACS buffer (0.2% BSA in PBS). Cells were incubated with anti-CD45.2 (BioLegend, clone 104), anti-H2K^b^ (BD Biosciences, clone AF6-88.5), and Zombie NIR (BioLegend, 423106) for 30 minutes at 4°C. Cells were washed twice, resuspended in FACS buffer, and analyzed on a Cytek Northern Lights Cytometer. For antibody information see Supplemental Table 1.

### Confocal microscopy analysis.

Mouse primary colonic epithelial cells (PCECs; C57-6047, Cell Biologics) were grown to 70% confluence in 4-well chamber slides (Nunc Lab-Tek II Chamber Slide) coated with a gelatin-based coating (Cell Biologics). Cells were either untreated or treated with LPS, CQ, or LPS plus CQ for 4 hours. Slides were sequentially stained with β2M (ab218230, Abcam) and LC3A/B (Cell Signaling Technology) antibodies and mounted in Prolong Gold (RI = 1.5). Fluorescence was imaged onto GaAsP detectors, using a Nikon A1 confocal scan head and ×60 1.4 NA oil objective, with identical image acquisition settings used for all samples.

### Cytokine ELISA.

Concentrations of IFN-γ, TNF-α, and IL-6 were measured in culture supernatants or in serum by ELISA with specific anti-mouse monoclonal antibodies using OptEIA ELISA Kits (BD Biosciences). Assays were performed according to the manufacturer’s protocol and read at 450 nm in a microplate reader (SpectraMax M2, Molecular Devices).

### Hepatocyte isolation.

Primary hepatocytes were isolated from mice 9–10 weeks of age via collagenase perfusion and Percoll gradient separation. Mice were anesthetized by intraperitoneal injection of ketamine. The liver was perfused by cannulation of the inferior vena cava with the hepatic portal vein as a drain. The liver was perfused first with prewarmed perfusion buffer containing Hanks balanced salt solution (HBSS, Gibco), 0.5 mM EDTA, and 25 mM HEPES, then with prewarmed digestion medium containing HBSS with Ca^2+^ and Mg^2+^ and phenol red, 25 mM HEPES, and 250 μg/mL Liberase Research Grade (MilliporeSigma). The liver was surgically removed, and hepatocytes were released into 10 mL of perfusion buffer and filtered through a 100 μm cell strainer. The suspension was then washed 2 times with 25 mL of warmed DMEM [+] 4.5 g/L glucose, l-glutamine [–] sodium pyruvate (Corning) and centrifuged at 50*g* for 2 minutes. Live hepatocytes were obtained after a 45% Percoll Plus gradient.

### Immunoprecipitation and Western blot.

Mouse PCECs (C57-6047, Cell Biologics) were grown to 70% confluence in 150 mm dishes. Cells were either untreated or treated with LPS, CQ, or LPS plus CQ for 4 hours. At time of harvest, cells were washed twice with warm PBS and then incubated with trypsin until they detached, followed by collection and pelleting. Pelleted cells were washed twice with cold PBS and then resuspended in ice-cold modified RIPA buffer (1 × 10^7^). Cells were then transferred to centrifuge tubes and gently mixed on a rocker at 4°C for 15 minutes. Supernatants were collected and precleared by incubation with 100 μL Protein A/G Magnetic Agarose Beads (Thermo Fisher Scientific) at 4°C for 10 minutes. After removal of magnetic agarose with a magnetic column, 10 μg β2M (ab218230, Abcam) antibody or control IgG was added to supernatants, and they were gently mixed on a rocker overnight. To capture immunocomplexes, 100 μL Protein A/G Magnetic Agarose Beads were then added to each sample, and they were gently mixed on a rocker overnight. Magnetic agarose was captured by a magnetic column and washed twice with cold PBS, and immunocomplexes were dissociated in boiled lysis buffer. Supernatants were then separated by 12.5% SDS-PAGE, transferred to membranes, and analyzed by immunoblotting with LC3A/B (Cell Signaling Technology) and β2M (ab218230, Abcam) antibodies. Immunoprecipitation (IP) against endogenous β2M was carried out in C57BL/6 primary mouse hepatocytes (C57-6224F, Cell Biologics), cardiac cells (C57-6228, Cell Biologics), and kidney cells (C57-6227, Cell Biologics). Adherent cells were washed twice with 10 mL of ice-cold PBS, harvested mechanically, and pelleted at 1,200*g* for 5 minutes at 4°C. Cells were lysed with IP lysis buffer (Mg2^+^-free PBS supplemented with 1% Triton X-100 and 1% Xpert Protease Inhibitor Cocktail [P3100-005, GenDEPOT]) for 10 minutes with end-over-end rotation at 4°C and centrifuged at 4,000*g* for 5 minutes at 4°C. A small fraction of the supernatant was collected for input material and stored at –80°C until SDS-PAGE separation. Then, the supernatant/cell lysate was mixed with either 4 μg of anti-β2M antibody (ab218230, Abcam) or normal rabbit IgG (2729S, Cell Signaling Technology) as a negative control in a total volume of 450 μL. Incubations with target antibody or isotype control were carried out for 1 hour 40 minutes with end-over-end rotation at 4°C, followed by incubation with 50 μL of protein G Dynabeads (10004D, Thermo Fisher Scientific) overnight at 4°C. The next day, protein G beads were washed once with 850 μL of ice-cold lysis buffer for 5 minutes with end-over-end rotation at 4°C, followed by a similar wash with 850 μL of ice-cold PBS supplemented with 1% Xpert Protease Inhibitor Cocktail. Protein complexes were eluted with 60 μL of elution buffer (50 mM Tris-HCl, pH 7.4, 1% SDS, 10 mM EDTA) by vortexing and application of 3 incubation cycles of 5 minutes at 65°C. IP protein complexes were resolved on a precast Any Kd Bio-Rad SDS-PAGE polyacrylamide gel (4569033, Bio-Rad) for 1 hour at 110 V and transferred for 7 minutes onto a PVDF membrane using a Trans-Blot Turbo transfer system (Bio-Rad). Western blot analysis against LC3B was performed with a rabbit monoclonal anti-LC3A/B antibody (12741, Cell Signaling Technology) diluted 1:1,000 in 1× Tris-buffered saline–Tween-20 (TBST) with 5% BSA, and incubated overnight at 4°C. The β2M protein was detected with a rabbit polyclonal anti-β2M antibody (13511-AP, Proteintech) diluted 1:2,000 in 1× TBST with 5% nonfat dry milk, and incubated overnight at 4°C. VeriBlot for IP Detection Reagent (ab131366, Abcam) was applied at 1:2,000 dilution in 1× TBST with 2.5% nonfat dry milk and was incubated for 2 hours 30 minutes at room temperature before visualization of the HRP-conjugated proteins with the ECL Clarity Western substrate (170-5060, Bio-Rad). Protein signals were captured on a Bio-Rad ChemiDoc MP imaging system.

### Cytotoxic T lymphocyte killing assay.

To obtain bone marrow–derived dendritic cells (BMDCs), bone marrow from B6 WT mice was isolated and cultured with GM-CSF for 7 days in vitro. The cells were harvested, and CD11c^+^ cells were isolated by magnetic separation with CD11c^+^ Microbeads Ultrapure (130-125-835, Miltenyi Biotec Inc.). BALB/c splenic T cells were isolated using CD90.2^+^ microbeads (130-049-101, Miltenyi Biotec Inc.) and cocultured with BMDCs at a ratio of 1:40 for 7 days. After coculture, activated T cells were isolated and used as effector cells. Primary hepatocytes were isolated for B6 WT or B6 Albumin-KO mice and used as target cells. 5 × 10^5^ target cells were incubated with 0.5 MBq of Na_2_^51^CrO_4_ (Cr51) (NEZ030001MC, PerkinElmer) for 2 hours at 37°C in a 5% CO_2_ atmosphere. After washing, 5 × 10^3^ labeled target cells were resuspended and added to triplicate wells at varying effector-to-target ratios and then incubated for 4 hours. Maximal and minimal Cr51 release was determined by addition of either Triton X-100 or medium alone to target cells, respectively.

### Statistics.

The Mann-Whitney *U* test was used for the statistical analysis of in vitro data, and Wilcoxon’s rank test was used to analyze survival data. For analyzing the changes within the sample, a paired 2-tailed *t* test was used. A *P* value less than 0.05 was considered statistically significant.

### Study approval.

Animals were cared for according to regulations reviewed and approved by the University Committee on Use and Care of Animals at the University of Michigan (PRO00009494), based on University Laboratory Animal Medicine guidelines. Mice were maintained at an Association for Assessment and Accreditation of Laboratory Animal Care International–accredited, specific pathogen–free animal facility at Baylor College of Medicine. The Institutional Animal Care and Use Committee at Baylor College of Medicine approved all experiments under protocol AN-8909.

### Data availability.

Raw data for the article are also available in the Supporting Data Values file.

## Author contributions

KOW, EL, and AT designed and performed experiments, analyzed the data, and wrote the paper. KOW is listed in first authorship position due to greater contribution in the writing process. LM, JLVN, YS, and LL performed experiments. CL performed experiments and histopathological analysis. DZ contributed reagents and helped analyze the data. PR designed experiments, analyzed the data, and wrote the paper.

## Supplementary Material

Supplemental data

Unedited blot and gel images

Supporting data values

## Figures and Tables

**Table 1:**
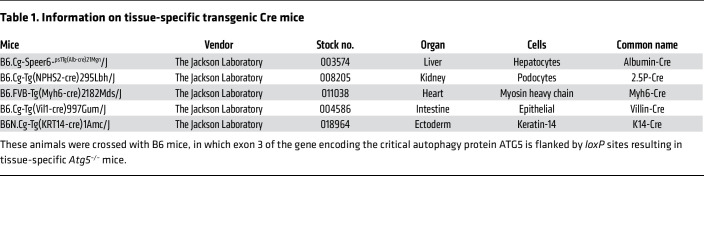
Information on tissue-specific transgenic Cre mice

## Version 1 03/01/2024

Electronic publication
