# Corrigendum to Autophagy differentially regulates tissue tolerance of distinct target organs in graft-versus-host disease models

**DOI:** 10.1172/JCI198274

**Published:** 2025-09-02

**Authors:** Katherine Oravecz-Wilson, Emma Lauder, Austin Taylor, Laure Maneix, Jeanine L. Van Nostrand, Yaping Sun, Lu Li, Dongchang Zhao, Chen Liu, Pavan Reddy

Original citation: *J Clin Invest*. 2024;134(5):e167369. https://doi.org/10.1172/JCI167369

Citation for this corrigendum: *J Clin Invest*. 2025;135(17):e198274. https://doi.org/10.1172/JCI198274

The authors recently became aware of missing funding information in the original publication. An updated Acknowledgments section is provided below:

This work was supported by NIH grants (R01HL152605, P01HL149633, and R01CA217156 to PR; 1R35-GM146762-01 to JLVN) and by the Cancer Prevention and Research Institute of Texas (RR220033 to PR and RR210013 to JLVN). JLVN is a CPRIT Scholar in Cancer Research.

The HTML and PDF versions of the paper have been updated.

The authors regret the error.

## Version 1 09/02/2025

Electronic publication

