# Supplementary material for: Autophagy differentially regulates tissue tolerance of distinct target organs in graft-versus-host disease models
Source: J Clin Invest. 2024 Mar 1;134(5):e167369. doi: 10.1172/JCI167369 (PMC10904048; doi:10.1172/JCI167369)
Supplement: Supplemental data [file jci-134-167369-s261.pdf]

Supplemental Figure 1

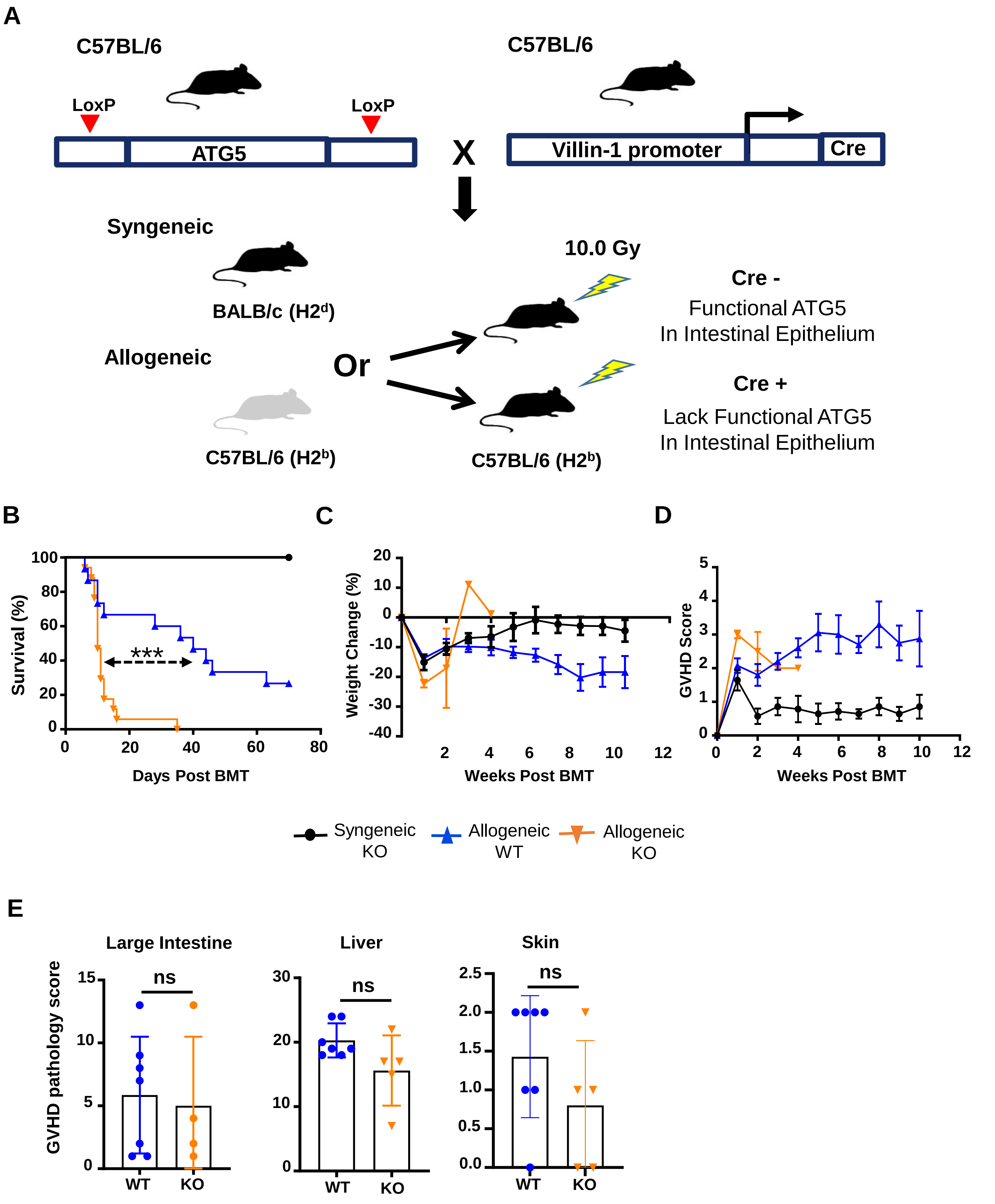

Supplemental Figure 1

F

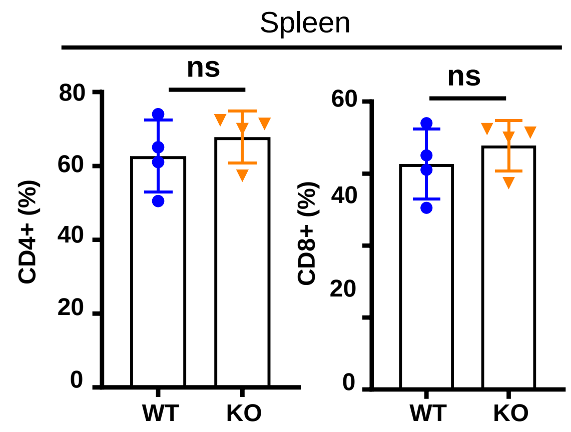

G

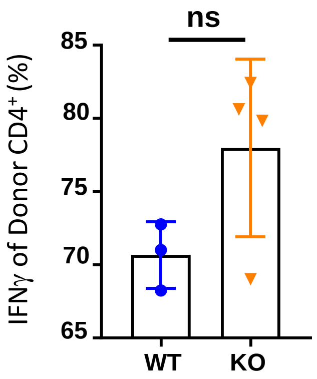

H

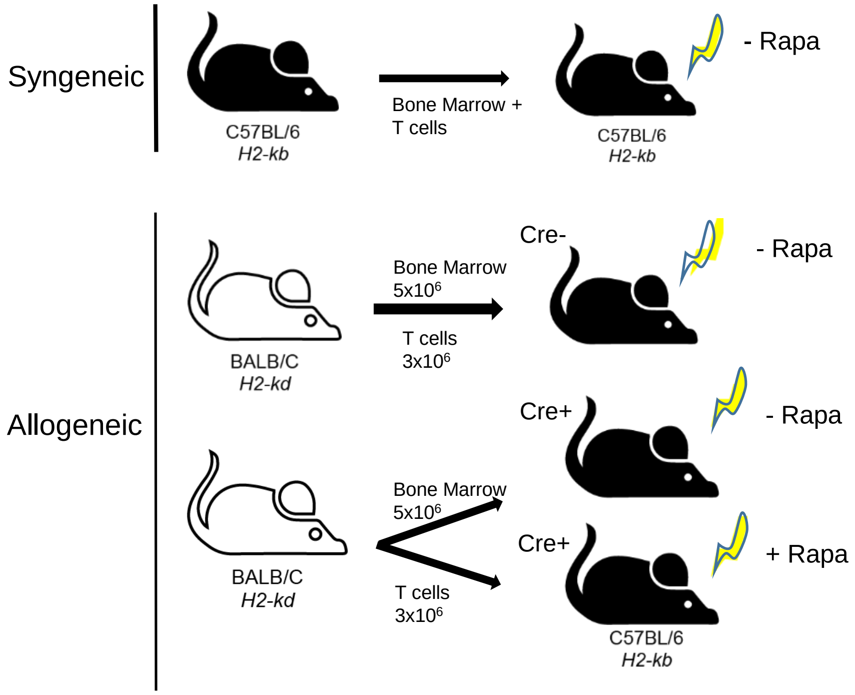

Supplemental Figure 1. Villin-Cre+;*Atg5*$^{-/-}$ mice show more severe GVHD post-allo-BMT in the absence of autophagy in the gut.

Schematic of mouse models used to inhibit autophagy in target and non-target GVHD tissues; strategy for intestinal *Atg5* knockout is shown (**A**). Villin-Cre+; *Atg5*$^{-/-}$ mice (B6 Villin-KO) and Villin-Cre-; *Atg55*$^{flox/flox}$ littermate controls (B6 WT) on a C57BL/6 background, were used as recipients in syn and MHC-matched but multiple minor antigen-mismatched allo-BMT using purified splenic T cells from B6C3H.sw mice. Mice were monitored weekly for survival (**B**), change in weight (**C**), and GVHD score (**D**). GVHD severity in large intestine, liver and skin on day 7 post allo-BMT (**E**). Total numbers of $CD4^+$ and $CD8^+$ T cells in B6 Villin-KO and B6 WT mice on Day 4 post-BMT from spleen measured by flow cytometry (**F**). Interferon (IFNγ) producing donor (H2-K^{d+}) $CD4^+$ splenic T cells in B6 WT and B6 Villin-KO mice at day 4 post-BMT, measured by flow cytometry (**G**). Schematic of experimental methodology for sirolimus (rapa) treatment in B6 Villin-KO mice (**H**). Supplemental Figure 1 **B-D** BMT data represents a combination of 2 independent experiments (Syn B6 WT, n = 4; Syn Villin-KO, n = 4; Allo B6 WT, n = 10; Allo Villin-KO, n = 10). **E** represents combined data from 2 independent experiments analysis on D7 post BMT (B6 WT, n=7; B6 Villin-KO, n=5). **F-G** represents experiment on D4 post Allo BMT (B6 WT, n=3-4; B6 Villin-KO n=4). Significance was determined using a log-rank (Mantel-Cox) test for survival data. Significance was determined using unpaired T test for weight and GVHD score. *P < 0.05, **P < 0.01, ***P < 0.001 and ****P < 0.0001.

Supplemental Figure 2

A

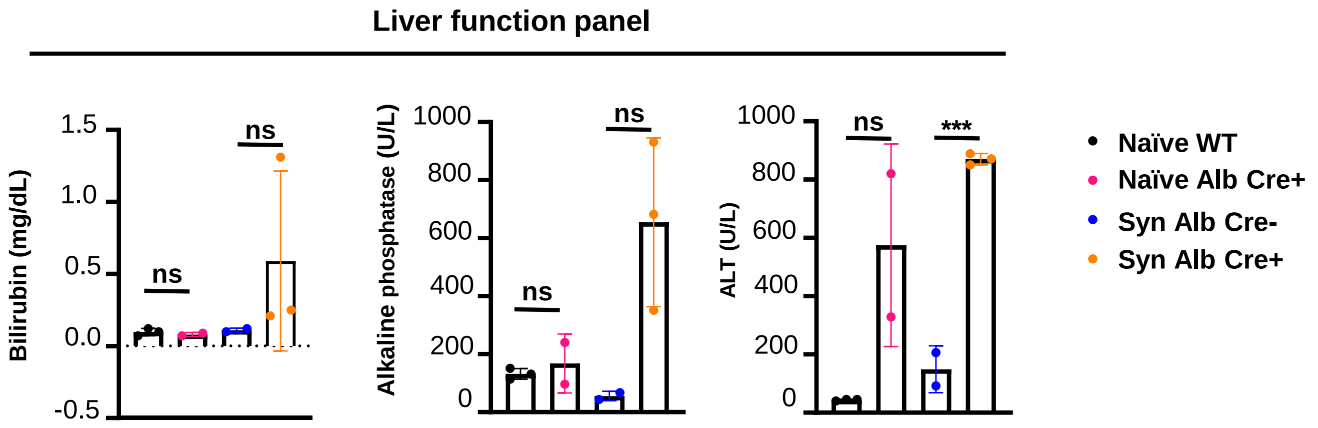

B

	Naïve WT	Naïve Cre+ ATG5$^{-/-}$	Syn Cre- ATG5$^{fl/fl}$	Syn Cre+ ATG5$^{-/-}$	Allo Cre- ATG5$^{fl/fl}$	Allo Cre+ ATG5$^{-/-}$
Bilirubin	*0.10 (± 0.03)*	*0.08 (± 0.01*	0.11 *(± 0.01)*	0.59 *(± 0.62)*	0.09 *(± 0.05)*	0.48 *(± 0.22)*
Alkaline Phosphatase	*132 (± 18.5)*	*168 (± 101.8)*	55.5 *(± 16.3)*	654.7 *(± 291)*	25 *(± 9)*	533.3 *(± 67.6)*
AST	*53 (± 3.6)*	*554 (± 100.4)*	627 *(± 35.4)*	295.3 *(± 175.5)*	512.3 *(± 152.9)*	394.3 *(± 113.6)*
ALT	*43.3 (± 2.9)*	*574 (± 347.2)*	149 *(± 80.6)*	870 *(± 19.5)*	142.3 *(± 70.6)*	780.0 *(± 15.4)*
Albumin	*3.80 (± 0.0)*	*3.1 (± 0.14)*	3.25 *(± 0.07)*	2.8 *(± 0.17)*	2.53 *(± 0.21)*	2.65 *(± 0.07)*

C

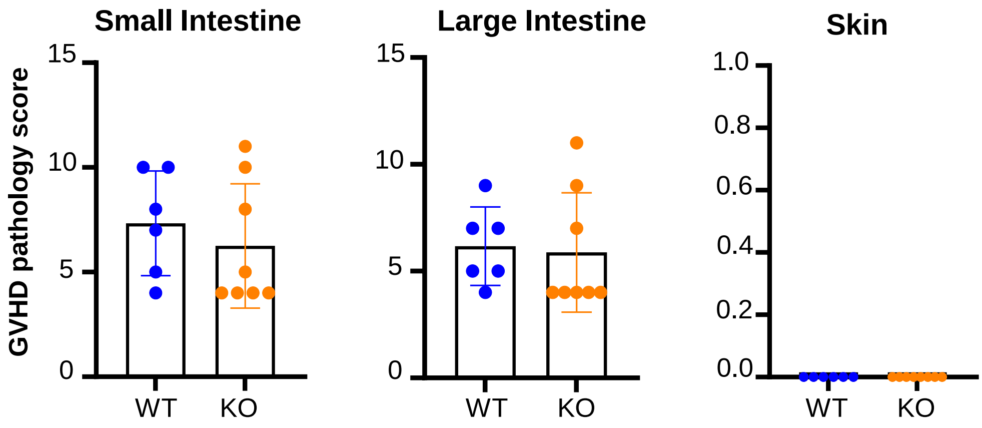

D

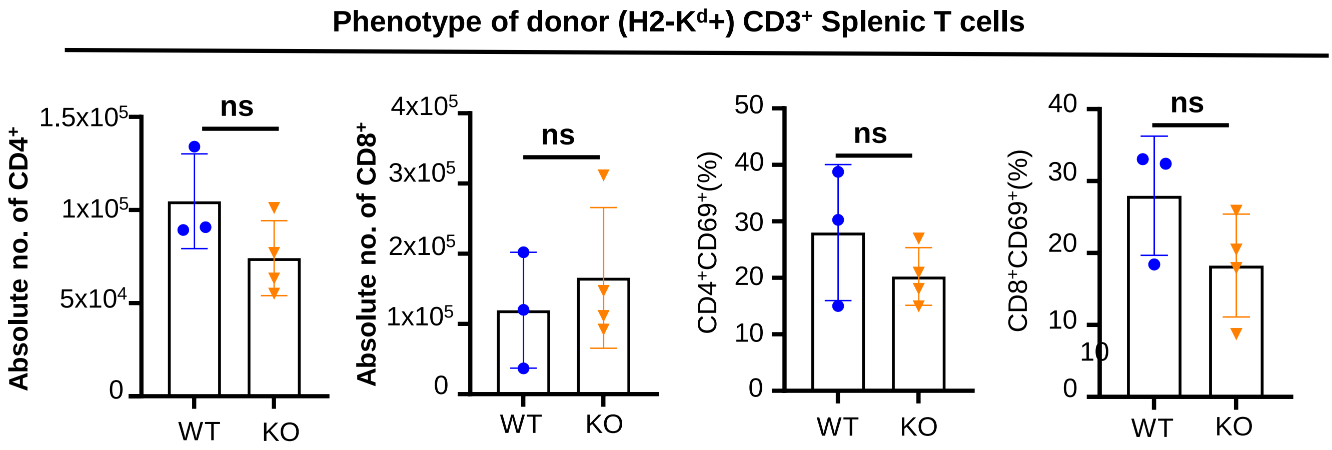
Phenotype of donor (H2-Kd+) CD3+ Splenic T cells
Absolute no. of CD4+
1.5x10^5
1x10^5
5x10^4
0
ns
WT
KO
Absolute no. of CD8+
4x10^5
3x10^5
2x10^5
1x10^5
0
ns
WT
KO
CD4+CD69+(%)
50
40
30
20
10
0
ns
WT
KO
CD8+CD69+(%)
40
30
20
10
0
ns
WT
KO

E

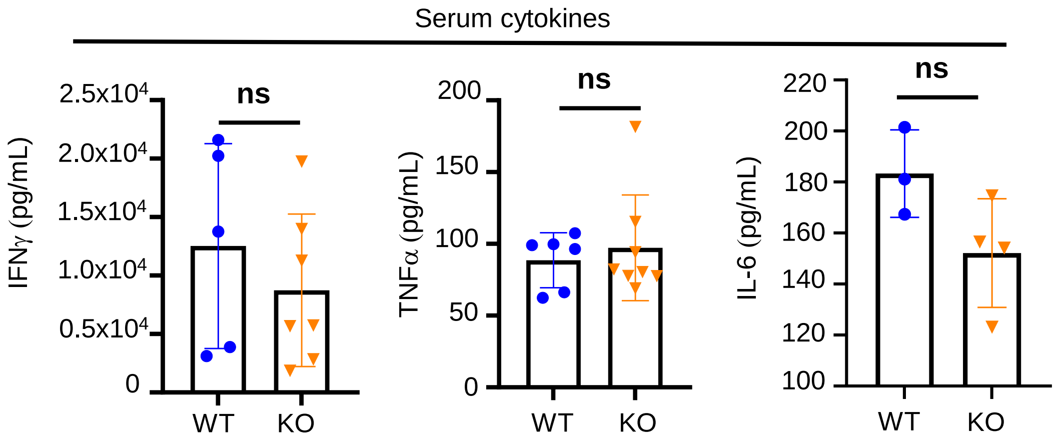
Serum cytokines
IFNγ (pg/mL)
2.5x10^4
2.0x10^4
1.5x10^4
1.0x10^4
0.5x10^4
0
ns
WT
KO
TNFα (pg/mL)
200
150
100
50
0
ns
WT
KO
IL-6 (pg/mL)
220
200
180
160
140
120
100
ns
WT
KO

Supplemental Figure 2. Albumin-Cre+; *Atg5*$^{-/-}$ mice show greater mortality post-allo-BMT in the absence of autophagy in the liver.

Liver function analysis by complete serum panel in B6 Albumin-KO, B6 WT, in either naïve or syn-BMT mice; Bilirubin, Alkaline Phosphatase and alanine aminotransferase (ALT) analysis (**A**). Table of complete serum panel values in naïve, syn- and allo-BMT in B6 Albumin-KO and B6 WT mice; Bilirubin, alkaline phosphatase, Aspartate Transaminase (AST), ALT, and Albumin (**B**). GVHD pathology scores at day 7 post-BMT for small intestine, large intestine, and skin (**C**). Phenotype of donor (H2-K^d+) $CD3^+$ splenic T cells at day 4 post-BMT, measured by flow cytometry; total numbers of $CD4^+$ and $CD8^+$ T cells and of $CD4^+CD69^+$ and $CD8^+CD69^+$ T cells in B6 Albumin-KO and B6 WT mice are shown (**D**). Concentration of serum cytokines on day 7 post-BMT in B6 Albumin-KO and B6 WT mice, measured by ELISA (**E**). **A-B** represents liver panel analysis on D7 post allo BMT (Naïve B6 WT, n=3; Naïve B6 Albumin-KO, n=2; Syn B6 WT, n = 2; Syn Albumin-KO, n = 3). **C** represents combined data from 2 independent experiments analyzed at day 7 post BMT (Allo B6 WT, n = 6; Allo B6 Albumin-KO, n = 8). **D** represents data comparing 2 groups (B6 WT, n = 3; Allo Albumin-KO, n = 4). **E** represents combined data from 2 independent experiments analyzed at day 7 post BMT (Allo B6 WT, n = 3-6; Allo Villin-KO, n = 4-8). Significance was determined using unpaired T test for weight and GVHD score. *P < 0.05, **P < 0.01, ***P < 0.001 and ****P < 0.0001.

A

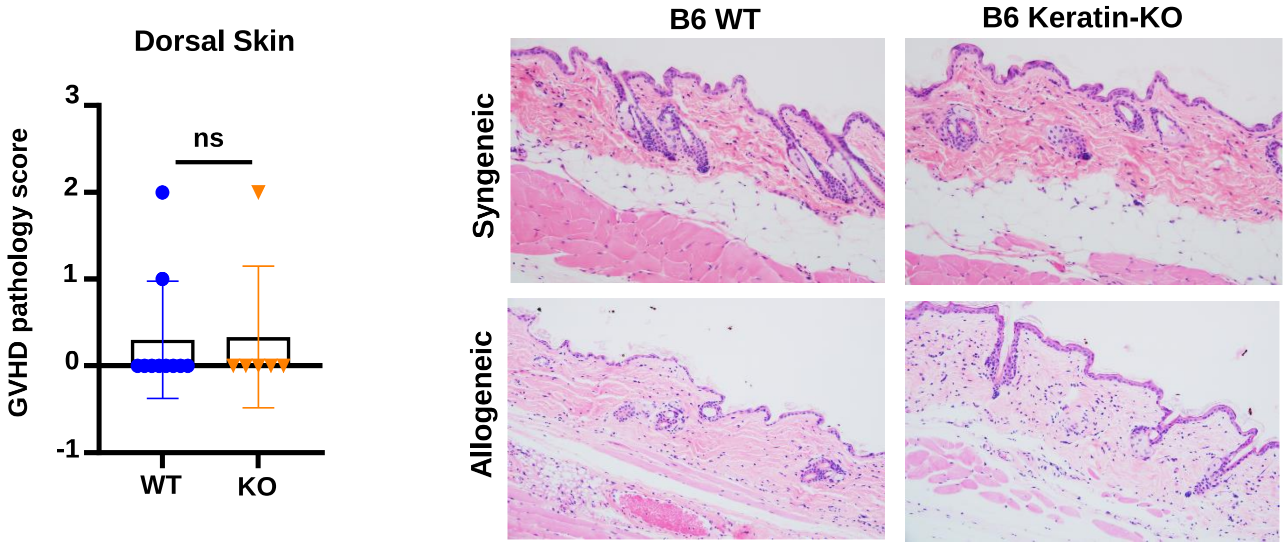
Dorsal Skin
GVHD pathology score
3
2
1
0
-1
ns
WT
KO
B6 WT
B6 Keratin-KO
Syngeneic
Allogeneic

B

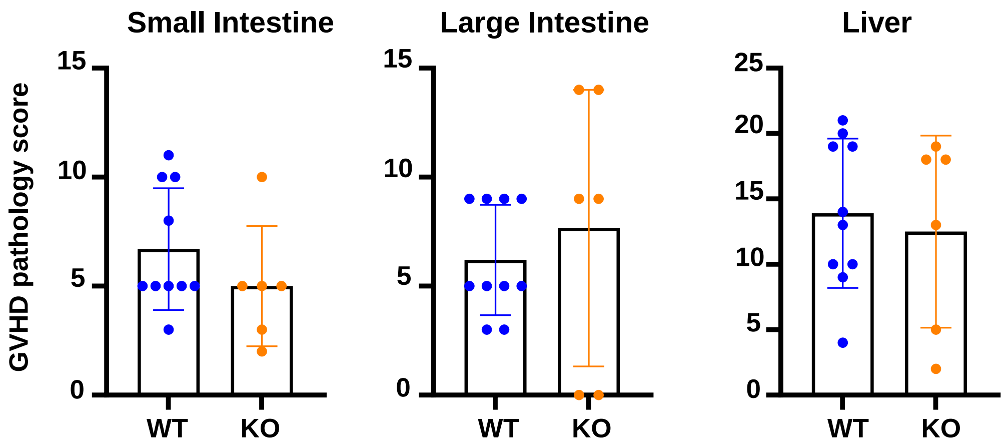
Small Intestine
Large Intestine
Liver
GVHD pathology score
15
10
5
0
25
20
WT
KO

Supplemental Figure 3. Keratin-Cre+; *Atg5$^{-/-}$* mice display no phenotype post-allo-BMT in the absence of autophagy in skin.

GVHD pathology scores and representative micrographs (original magnification, x20) of H&E staining of dorsal skin tissue sections from B6 Keratin-KO and B6 WT littermate controls biopsied at day +75 post-BMT (**A**). GVHD pathology scores for small intestine, large intestine, and liver on day 75+ post allo-BMT (**B**). Supplemental Figure 3 **A-B** represents a combination of 2 independent experiments (Allo B6 WT, n = 10; Allo B6 Keratin-KO, n = 6). Significance was determined using unpaired T test for GVHD score. *$P < 0.05$, **$P < 0.01$, and ***$P < 0.001$.

Supplemental Figure 4

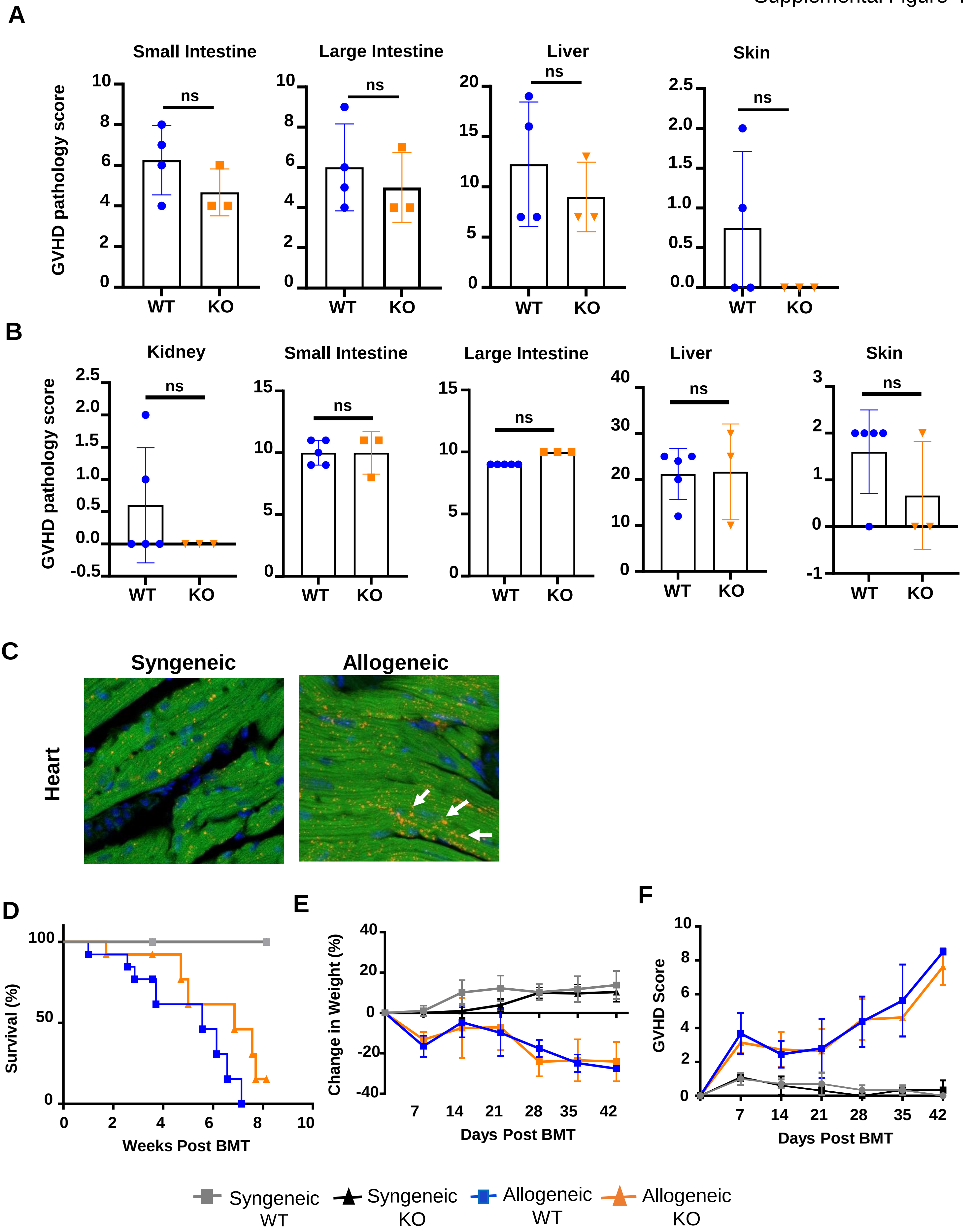

Supplemental Figure 4. Non-GVHD target tissues mouse models show no phenotypic differences in the absence of autophagy.

Pathology score of GVHD severity in B6 Podocin-KO and B6 WT mice at day 7 post-BMT in GVHD target tissues; Intestine, liver and skin (**A**). Pathology score of GVHD severity in B6 Podocin-KO and B6 WT mice at days 28-56 days post-BMT in the kidney, SI, LI, liver, and skin (**B**). Verification of autophagy induction on day 3 post-BMT in heart from CAG-RFP-EGFP-LC3 syngeneic (syn) and allogeneic (allo) transplanted animals. Co-expression of eGFP (green) and RFP (red), indicated by yellow punctate staining, occurs when LC3 is in the cytoplasm and autophagosomes. An increase in red punctate staining, marked by arrowheads, indicates LC3 in autophagolysosomes (**C**). Myh6-Cre^{+}; *Atg5*$^{-/-}$ mice (B6 Myocyte-KO) and Myh6-Cre-; *Atg5*$^{flox/flox}$ littermate controls (WT) on a C57BL/6 background were used as recipients in syn and MHC-mismatched allo-BMT. Mice were monitored weekly for survival (**D**), change in weight (**E**) and GVHD score (**F**) for B6 Myocyte-KO and B6 WT mice after syn and allo-BMT. **A** represents an experiment with data comparing 2 groups (Allo B6 WT, n = 4; Allo Podocin-KO, n = 3). **B** represents remaining mice after 4-8 weeks survival studies with data comparing 2 groups (Allo B6 WT, n = 5; Allo Podocin-KO, n = 3). **D-F** represents a combination of 2 independent experiments (Syn, n=10; Allo B6 WT, n = 13; Allo B6 Myocyte-KO, n = 13). Significance was determined using a log-rank (Mantel-Cox) test for survival data. Significance was determined using unpaired T test for weight and GVHD score. *P < 0.05, **P < 0.01, and ***P < 0.001.

Supplemental Figure 5

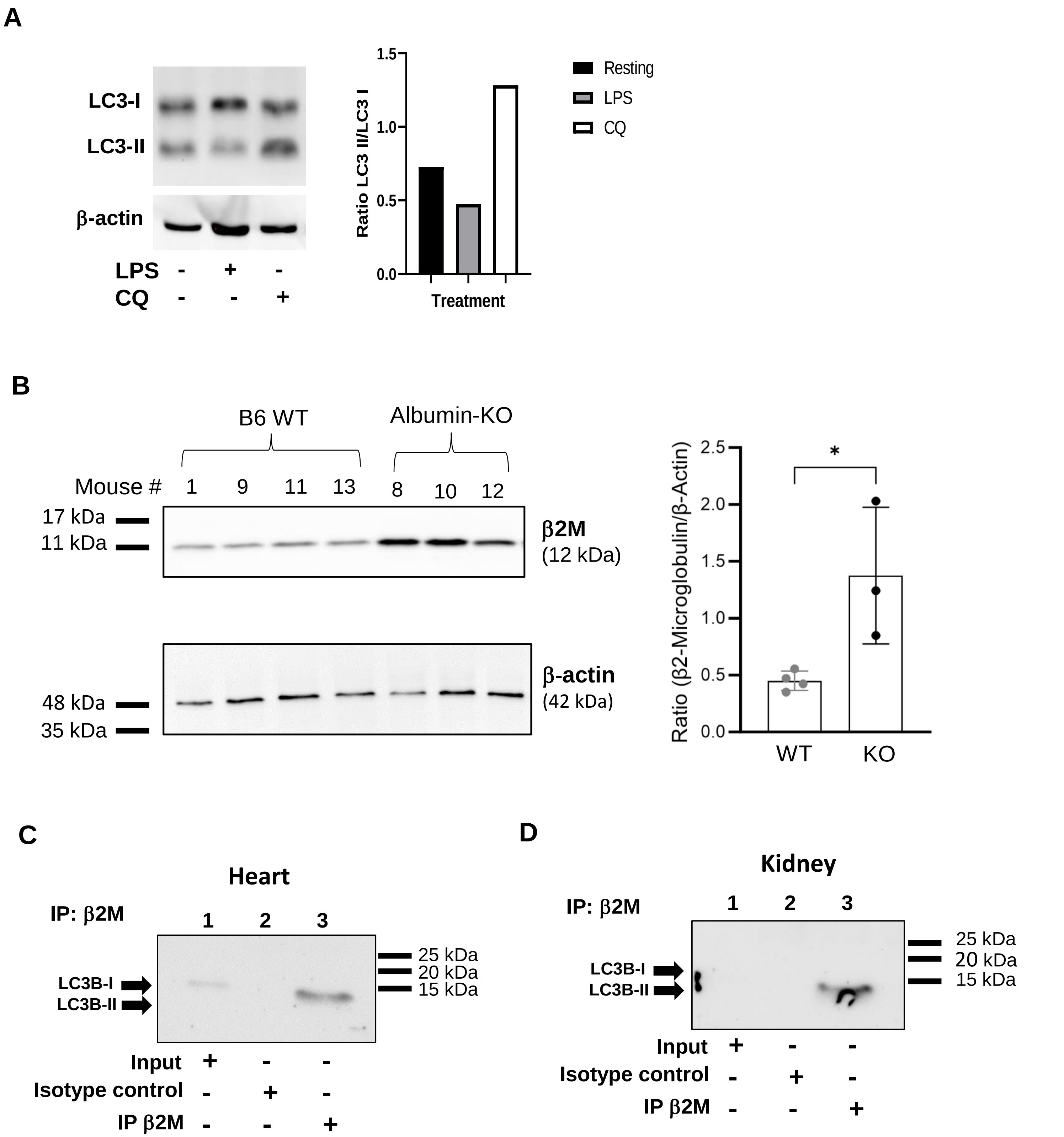

Supplemental Figure 5. IECs from Villin-Cre+;*Atg5*$^{-/-}$ mice show increased levels of MHC-1 compared to WT cells.

Whole-cell lysates of PCECs treated with either LPS or CQ and untreated control cells were analyzed by western blot (WB) to measure LC3A/B expression (**A**). Lysates from untreated primary mouse hepatocyte cells were analyzed by WB for MHC-I levels (**B**). Lysates from untreated primary mouse heart cells (**C**) primary mouse kidney cells (**D**) were immunoprecipitated (IP) with MHC-I (β2M) antibody and analyzed by western blotted with LC3B antibody. **A** is a representative graph of 3 independent experiments. **B** represents an experiment with data comparing 2 groups (Allo B6 WT, n = 4; Allo Albumin-KO, n = 3). **C-D** is representative of 1 independent experiment each. Significance was determined using unpaired T test for weight and GVHD score. *$P < 0.05$, **$P < 0.01$, and ***$P < 0.001$.

Supplemental Table 1.

Antibody	Vendor	Catalog Number
**CD4-APC**	Biolegend	100412
**CD8-PE**	Biolegend	100708
**CD62L-PerCP/Cy5.5**	Biolegend	104432
**CD69-FITC**	Biolegend	104505
**Foxp3-PE**	Biolegend	126404
**IFNg- APC-Cy7**	Biolegend	505850
**H2kb-PE**	Biolegend	116508
**CD326-APC**	Biolegend	118214
**CD326-FITC**	Biolegend	118208
**I-A/I-E-APC-Cy7**	Biolegend	107628
**β2M**	Abcam	ab218230
**LC3B**	Novus Biologicals	NB100-2331
**CD45.2**	Biolegend	109813
**H2kb**	BD Bioscience	566240
**Zombie NIR Fixable Viability Kit**	Biolegend	423105
**LC3 A/B**	Cell Signaling	12741
**Actin**	Abcam	ab8224
**Rabbit IgG Isotype Control**	Cell Signaling	2729S
