# Supplementary material for: Autophagy differentially regulates tissue tolerance of distinct target organs in graft-versus-host disease models
Source: J Clin Invest. 2024 Mar 1;134(5):e167369. doi: 10.1172/JCI167369 (PMC10904048; doi:10.1172/JCI167369)
Supplement: Unedited blot and gel images [file jci-134-167369-s262.pdf]

Full unedited gel for Figure 5E

Intestinal Epithelial

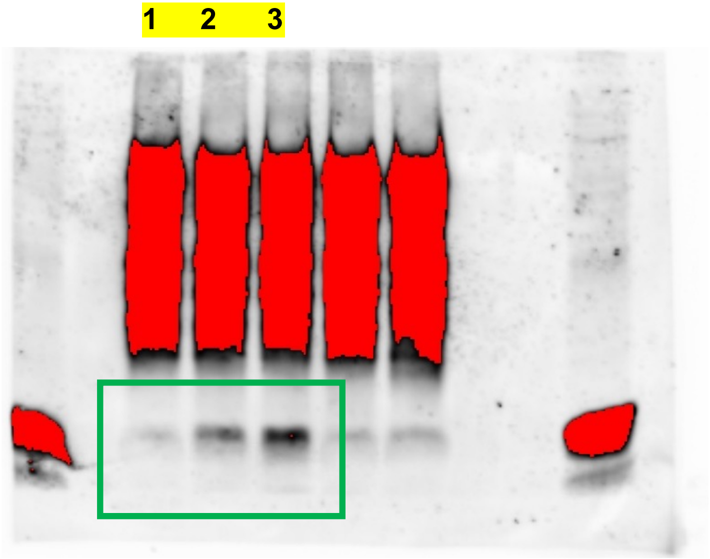

Rabbit monoclonal anti-LC3A/B antibody (Cell Signaling, # 12741)

Full unedited gel for Figure 5F

Hepatocyte

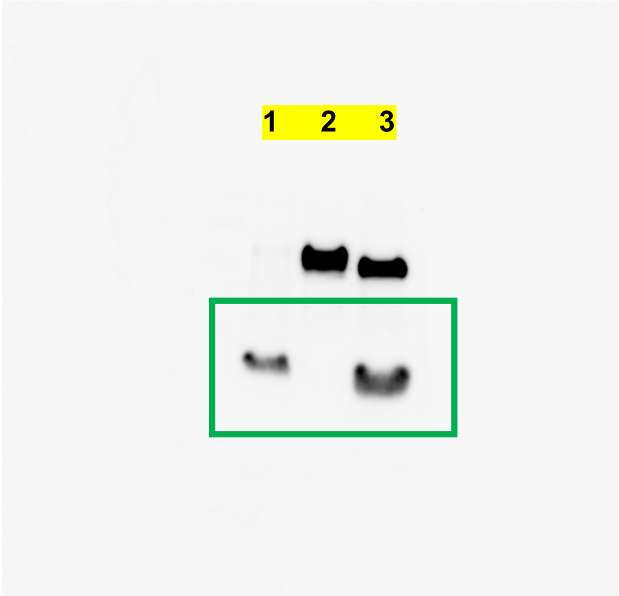

Rabbit monoclonal anti-LC3A/B antibody (Cell Signaling, # 12741)

Full unedited gel for Supplemental Figure 5A

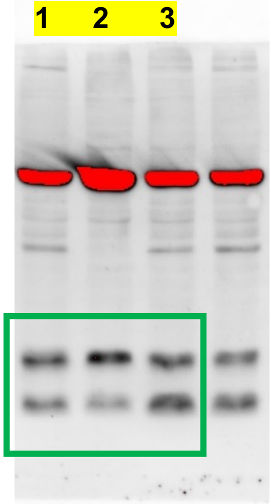

Rabbit polyclonal anti-LC3A/B antibody (Novus, Cat #NB100-2331)

Full unedited gel for Supplemental Figure 5A

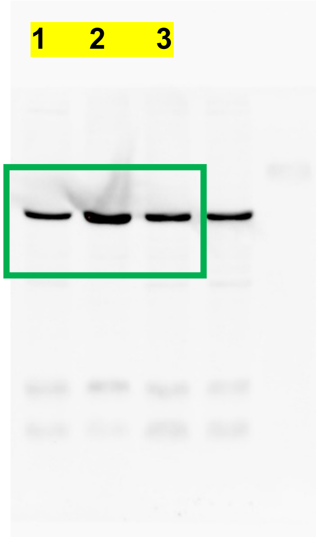

Mouse monoclonal beta-actin antibody (Abcam, # ab8224)

Full unedited gel for Supplemental Figure 5B

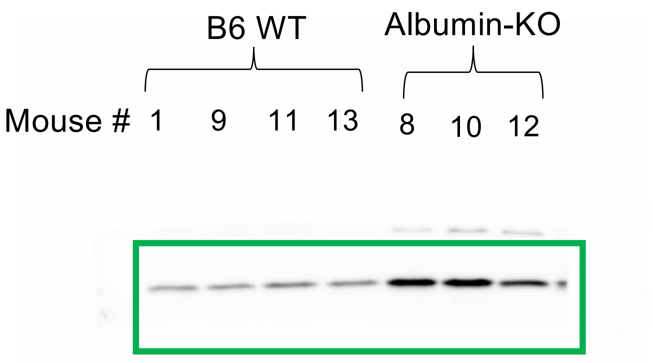

Rabbit monoclonal β2-microglobulin antibody (Abcam, # ab218230)

Full unedited gel for Supplemental Figure 5B

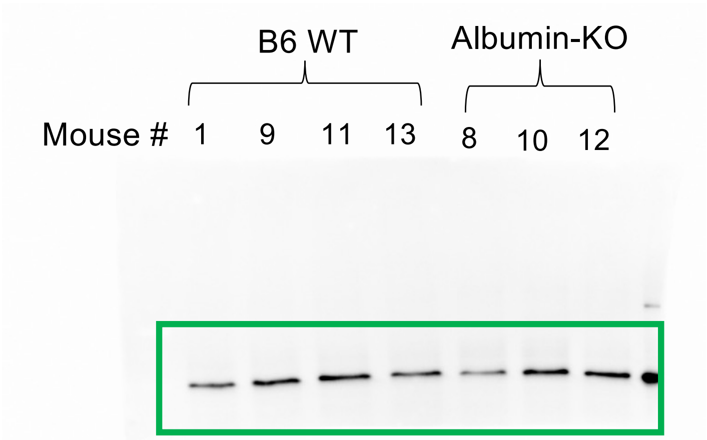

Mouse monoclonal beta-actin antibody (Abcam, # ab8224)

Full unedited gel for Supplemental Figure 5C

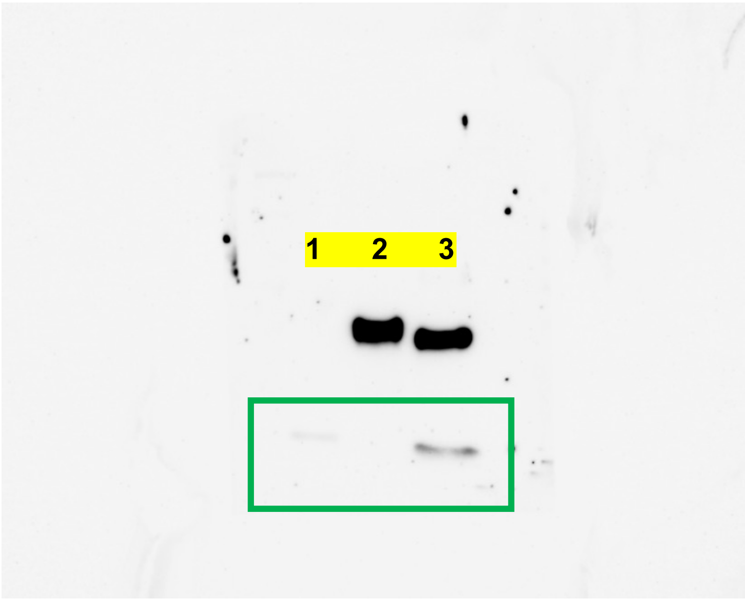

Rabbit monoclonal anti-LC3A/B antibody (Cell Signaling, # 12741)

Full unedited gel for Supplemental Figure 5D

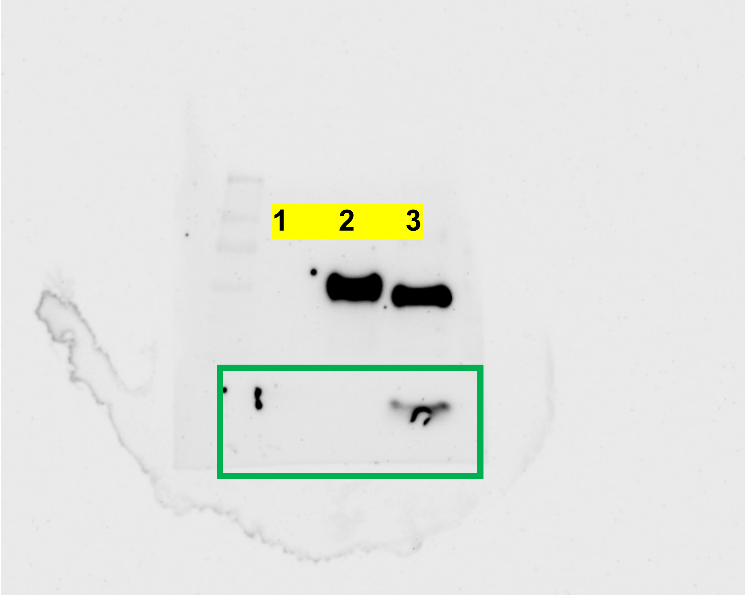

Rabbit monoclonal anti-LC3A/B antibody (Cell Signaling, # 12741)
